# Transcriptional profiling of corneal stromal cells derived from patients with keratoconus

**DOI:** 10.1038/s41598-019-48983-8

**Published:** 2019-08-29

**Authors:** Rabab Sharif, Mariam L. Khaled, Tina B. McKay, Yutao Liu, Dimitrios Karamichos

**Affiliations:** 1 Department of Cell Biology, University of Oklahoma Health science Center, Oklahoma City, Oklahoma 73104 USA; 2 Department of Cellular Biology & Anatomy, Augusta University, Augusta, GA 30912 United States; 3 Schepens Eye Research Institute and Department of Ophthalmology, Harvard Medical School, Boston, MA 02114 USA; 4 Department of Ophthalmology/Dean McGee Eye Institute, University of Oklahoma Health Science Center, Oklahoma City, Oklahoma 73104 USA

**Keywords:** Mechanisms of disease, Diagnostic markers, Corneal diseases

## Abstract

Keratoconus (KC) is a multi-factorial corneal ectasia with unknown etiology affecting approximately 1:2000 people worldwide. Dysregulated gene expression, using RNA-Seq technology, have been reported in KC corneal tissue. However, the differential expression of genes, in KC corneal stromal cells have been widely ignored. We utilized mRNA-Seq to analyze gene expression in primary human corneal stromal cells derived from five non-Keratoconus healthy (HCF) and four Keratoconus (HKC) donors. Selected genes were further validated using real time PCR (RT-PCR). We have identified 423 differentially expressed genes with 187 down- and 236 up-regulated in KC-affected corneal stromal cells. Gene ontology analysis using WebGestalt indicates the enrichment of genes involved in cell migration, extracellular matrix, adherens junction, and MAPK signaling. Our protein-protein interaction network analysis identified several network seeds, such as EGFR, NEDD4, SNTA1, LGALS3BP, HSPB1, SDC2, MME, and HIF1A. Our work provides an otherwise unknown information on the transcriptional changes in HKCs, and reveals critical mechanisms of the cellular compartment. It also highlights the importance of human-based *in vitro* studies on a disease that currently lacks strong biomarkers and animal models.

## Introduction

Keratoconus (KC) is a multi-factorial, complex, idiopathic disease of the cornea that leads to corneal thinning, corneal scarring, and the hallmark “cone-like” shape of the cornea^1^. Corneal epithelium iron deposition and Descemet membrane rupture are also seen in KC^2^. The disease usually affects both eyes and can deteriorate very fast leading to potentially blinding conditions to young people, if left untreated. KC affects approximately 1:400 people worldwide and typically begins during puberty, until its arrest in the third or fourth decade^3^. The reason for this arrest remain a mystery.

KC has a substantial financial burden associated with it, post-diagnosis. Though the monetary costs associated with KC are apparent, effects on more subjective areas, such as quality of life and job outlook, are harder to compute. Since KC is a progressive visual disorder, a diagnosis of KC is considered disqualifying for securing various jobs as well as joining the military^4^, regardless of best-corrected or even uncorrected visual acuity. Various studies have shown that individuals with KC have lower quality of life that stems from the lack of quality corrected vision^5–7^. The National Eye Institute’s Visual Function Questionnaire data from over 1,100 KC patients over a 7-year period identified a significant decline in quality of life in KC patients^8^. Therefore, efforts to improve methods of treatment are critical.

KC has been associated with various genetic and other exogenous degenerative factors^9,10^. However, despite significant advancements, the pathophysiology of KC remains poorly understood. A lack of an animal model has severely hampered our progress in unravelling the KC roots. Except for one case report of KC in a rhesus monkey^11^, KC has not been reported in animals. In addition, we have yet to see the development of a transgenic animal with the KC phenotype. These limitations are the main reason that key molecular mechanisms responsible for the disease onset and progression have not been identified. Thus, it is urgent to develop a global understanding of how changes occur, in KC, at the cellular level.

Gene expression profile using disease-affected tissues have been applied to identify disease-related genes and pathways using different high throughput technologies, including microarray and next generation sequencing (NGS). Using PCR-based subtractive hybridization, Wentz-Hunter *et al*. (2001) identified elevated expression of HSP90, decorin, fibronectin, ferritin heavy chain, and keratocan in KC-affected corneal stromal layer when compared to the normal/healthy stromal layer^12^. More gene expression studies using whole cornea, epithelium, or tears from individuals with or without KC have identified a number of differentially expressed genes and KC-related pathways^13–24^. Recently, three RNA-Seq-based expression profiling studies using whole cornea or corneal epithelium have identified many differentially expressed genes involved in TGFβ, WNT, and PI3K/AKT signaling in KC pathogenesis^13,21,25^.

We have previously shown that human corneal stromal cells isolated from KC patients, termed HKCs, maintain characteristics of the disease phenotype *in vitro* and secrete and assemble a thinner extracellular matrix (ECM) high in the pro-fibrotic marker, collagen type III, compared to their healthy counterparts^26,27^. Furthermore, consistent with tear analysis of KC patients *in vivo*^28–30^, we have found that HKCs maintain altered cellular metabolism^31^ correlating to increased oxidative stress^32^. In this study, for the first time, we have investigated the transcriptome profiles of primary human corneal stromal cells from Healthy and KC donors using RNA-Seq to identify global changes in transcript levels that may contribute to the disease phenotype. Our bioinformatics analysis and RT-PCR validation implicated the potential involvement of genes in several KC-related pathways. If *in vitro* studies are to lead the way towards future KC therapeutic targets, it is critical to examine the primary corneal cells used. Our study provides, otherwise unknown, information to our understanding of the disease mechanisms.

## Methods

### Ethics and inclusion criteria

Primary human corneal stromal cells were isolated from five healthy/normal and four KC individuals. The Institutional Review Board at the University of Oklahoma Health Science Center – Dean McGee Eye Institute approved the protocol and studies, prior to initiation. This study adhered to the tenets of the Declaration of Helsinki. Prior to collection of corneas, written permission was obtained from all subjects following corneal transplantation. None of the KC donors was from a vulnerable population and all donors or next of kin provided written informed consent that was freely available. Patients who had any other ocular or systemic disease, or previously underwent collagen crosslinking, were excluded from this study. All donors were examined comprehensively using Pentacam HR, refraction, and slit lamp to confirm KC diagnosis. Stabilization of corneal thinning or KC progression was not reported by the clinician prior to tissue isolation and remains unknown.

Healthy corneal tissues with no history of ocular or systemic diseases was provided by, the National Development and Research Institutes (NDRI).

### Cell isolation and expansion

Primary corneal stromal fibroblasts were isolated as previously described^33,34^. KC corneal tissue was provided from the Dean McGee Eye Institute in Oklahoma City, OK. Corneal stromal cells were isolated, by removing both the epithelial and endothelial layers with a sterile surgical scalpel. Tissues were then cut into small pieces (~2 × 2 × 2 mm), and incubated in sterile flasks to promote adhesion. Explants were supplemented with EMEM containing 10% fetal bovine serum (FBS, Atlanta biologicals, Flowery Branch, GA) and antibiotic/antimycotic (anti/anti, Life Technologies, Grand Island, NY), and incubated at 37 °C/5% CO_2_ for 2–3 weeks until cells migrated from the explant (~70–80% confluence).

Both Healthy Corneal Fibroblasts (HCFs) and Human Keratoconus cells (HKCs) were cultured on T75 tissue culture flasks. Cells were seeded at 5,000 cells/cm^2^ and maintained at 37 °C with 5% CO_2_ in a humidified incubator. Cells used in all experiments were between 2^nd^ and 4^th^ passages.

### RNA extraction

Total RNA was extracted using the Ambion RNA mini extraction kit (Ambion TRIzol® Plus RNA Purification Kit: Life technologies, Carlsbad, CA). Briefly, TriReagent® (Life Technologies Corporation, Carlsbad, CA), was added to the cell layer after aspiration of medium from the culture and brief washing with Phosphate Buffered Saline (PBS). The cellular layer was mechanically disrupted from tissue culture plate using gentle pipetting. Phase separation was conducted with chloroform and the total RNA contained in the aqueous phase was purified using RNeasy® mini kit column (QIAGEN, Hilden, Germany), according to the manufacturer’s protocol. Three extractions were carried out for each sample and pooled at the end of the RNeasy protocol. The purity and quantity of total RNA were evaluated using an ultraviolet spectrometer (CLARIOstar, BMG LABTECH, Cary, NC).

### RNA-Seq and bioinformatics analysis

RNA sequencing library was prepared using TruSeq RNA Library Prep kit, from Illumina with sample-specific indexes at the Oklahoma Medical Research Foundation (OMRF) Genomics facility. Briefly, 1 µg of total RNA was used to purify and fragment mRNA, followed by first and second strand cDNA synthesis. After purification and ligation of Illumina adapters and sample-specific indexes, sequencing libraries were validated using Agilent DNA 100 kit with Agilent Bio analyzer 2100. The libraries were normalized, and pooled for sequencing, with Illumina NextSeq 550 system using the paired end 75 bp with the high-output kit.

The data analysis for RNA-Seq was done using our established pipeline as described previously^13^. Briefly, after quality check and quality control with all the sequencing reads, demultiplexed reads were aligned by TopHat in paired-end reading with the approximation of the median library size^13^. Counts of sequencing reads were normalized using Cufflinks in fragments per kilo bases and millions reads (FPKM)^13^. Normalized sequencing read counts were analyzed by Cuffdiff^13^, with a transcript file from Ensembl database for the annotations at the gene as well as the isoform levels in a group comparison manner such as 4 cases vs 5 controls in this experimental design. Missing expression data in specific samples was replaced with a value of 0.001 to enable the differential analysis between cases and controls. Without such replacement of missing data, the related mRNAs would have been excluded from the differential expression analysis. In the final gene list with the positions at the corresponding chromosomes, it showed comparative fold changes and adjusted P values (false discovery rate, FDR) through the resulting analysis of four cases vs. five controls. Differentially expressed genes were defined to have an FDR value ≤ 0.05 and a |fold change| ≥ 2.

The differentially expressed genes were loaded into WEB-based Gene Set Analysis Toolkit (WebGestalt)^35,36^, for bioinformatics analysis to identify enriched gene ontologies, pathways, network modules, and associations with phenotypes/disease/drug. Potential targeted miRNAs were identified for the differentially expressed genes using WebGestalt.

### Validation using Real-time PCR

To validate the differential expression of selected genes, we used RT-PCR with all the donors/samples, as previously described^32,37,38^. The cDNA synthesis was followed using a SuperScript™ III First-Strand Synthesis SuperMix (Invitrogen, Carlsbad, CA) according to the manufacturer’s protocol. The TaqMan gene expression assays (Applied Biosystems, Foster City) for GAPDH (Hs99999905_m1) and 18S (Hs99999901_s1) were used as the reference assays to normalize target gene expression. We selected the following genes for validation: KRT7 (Hs00559840_m1), MME (Hs00153510_m1), ANKRD1 (Hs00173317_m1), ERG1 (Hs00152928_m1), IL1B (Hs01555410_m1) CXCL1 (Hs00236937_m1), and GDF15 (Hs00171132_m1). Furthermore, 10 ng of cDNA was used for initiating the PCR reaction for a 20-µl reaction mixture containing our desired probes and the TaqMan Fast Advanced MasterMix (Applied Biosystems, Life technologies, Foster City, CA). Amplification of samples was performed using the StepOnePlus^TM^ real-time PCR system (Life Technologies) in accordance with the manufacture’s protocol. Graph Pad Prism 7 and MS-Excel were used for data analysis.

### Statistical analysis

GraphPad Prism 7.02 was used to determine statistical significance using ANOVA or t-test, where appropriate. A p ≤ 0.05 was considered statistically significant.

## Results

### Clinical phenotypes

Our study included HCF cells from 5 postmortem donors (3 males/2 females) with postmortem delay less than 24 hours. All four HKC cells were derived from surgically removed keratoconic corneas immediately after surgery. It included 1 male and 3 females. The average age was 47.2 years for controls and 60.8 years for cases.

### HCFs and HKCs – transcriptional profiles

We performed RNA-Seq with primary human corneal stromal fibroblast cells derived from five unaffected controls (HCFN21, HCFN20, HCFN23, HCFN22, and HCFN4) and four KC patients (HKCWV1, HKCWV2, HKCDM1, and HKCDM2). Individual information for all the samples is provided in Table 1.

**Table 1:** Clinical phenotypes of the corneal donors.

Sample ID/code	Age (years)	Gender	Cause of Death
**HCF** N4	35	Female	Aspiration
**HCF** N20	26	Male	Head trauma
**HCF** N21	63	Male	MI (Myocardial infarction)
**HCF** N22	69	Male	ESRD (end stage renal disease)
**HCF** N23	43	Female	Head trauma/ICH (intracranial hemorrhage)
**HKC** Wu1	62	Female	Corneal transplant*
**HKC** Wu2	69	Male	Corneal transplant*
**HKC** DM1	44	Female	Corneal transplant*
**HKC** DM2	68	Female	Corneal transplant*

*These donors were Keratoconus patients with corneal transplantation.

All nine RNA samples were sequenced with 33.7–47.9 million paired-end reads of 75 nucleotides. All the samples had at least 30 million paired sequence reads aligned. Sequence reads with multiple alignments were removed during quality control process. Out of the total 15,159 genes, 11,540, 11,637, 11,568, 11,577, and 11,740 genes were expressed in the individual HCF cells with FPKM ≥ 1.0 and 9,870 genes were expressed in all five primary HCF cells with FPKM ≥ 1.0. 11,791, 11,768, 11,751, 12,122, and 11,544 genes were expressed in the individual HKC cells with FPKM ≥ 1.0 and 9,923 genes were expressed in all five primary HKC cells with FPKM ≥ 1.0. With all corneal stromal fibroblast cells from 10 donors, 9,302 genes were expressed with FPKM ≥ 1.0.

Our differential analysis using Cuffdiff identified 423 differentially expressed genes in HKC cells with at least 2 fold change and false discovery rate q value ≤ 0.05 (supplemental Table 1). There were 187 down-regulated and 236 up-regulated genes in KC-affected corneal stromal cells. After applying a minimum expression of 10 normalized reads in either cases or controls, a total of 208 genes (121 down and 87 up-regulated) were differentially expressed in HKC fibroblast cells (supplemental Table 2). The top 20 up- and down-regulated genes with largest fold changes are listed in Table 2.

**Table 2:** Top20 up- and down-regulated genes with the largest fold change in keratoconic corneal stromal cells.

Gene name	Gene Description	Normalized Average Expression (FPKM)		Fold change	FDR value
		Controls (n = 5)	KC (n = 4)		
ANKRD1	ankyrin repeat domain 1 (cardiac muscle)	119.62	0.28	−428.03	3.88E-03
AQP1	aquaporin 1 (Colton blood group)	17.25	0.12	−145.47	3.88E-03
MYH11	myosin, heavy chain 11, smooth muscle	17.91	0.14	−130.62	4.16E-02
MRVI1	murine retrovirus integration site 1 homolog	17.32	0.15	−117.10	3.88E-03
CRYAB	crystallin, alpha B	1632.04	14.65	−111.37	3.88E-03
A2M	alpha-2-macroglobulin	17.67	0.16	−108.64	3.88E-03
OXTR	oxytocin receptor	52.23	0.65	−80.61	9.23E-03
ACTA2	actin, alpha 2, smooth muscle, aorta	4845.11	65.95	−73.47	3.88E-03
SCUBE3	signal peptide, CUB domain, EGF-like 3	10.72	0.15	−69.63	3.88E-03
LGI4	leucine-rich repeat LGI family, member 4	13.20	0.25	−53.71	1.37E-02
PDLIM3	PDZ and LIM domain 3	20.99	0.46	−45.58	3.88E-03
ITGA7	integrin, alpha 7	91.90	2.11	−43.46	3.47E-02
MCAM	melanoma cell adhesion molecule	75.51	1.74	−43.32	3.88E-03
COL5A3	collagen, type V, alpha 3	16.03	0.40	−40.38	3.88E-03
RGS5	regulator of G-protein signaling 5	10.16	0.26	−39.21	9.23E-03
ID4	inhibitor of DNA binding 4, dominant negative helix-loop-helix protein	34.13	0.89	−38.35	3.88E-03
LMCD1	LIM and cysteine-rich domains 1	36.38	1.00	−36.34	3.88E-03
SGCA	sarcoglycan, alpha (50 kDa dystrophin-associated glycoprotein)	34.86	1.03	−33.81	3.88E-03
HSPB7	heat shock 27 kDa protein family, member 7	117.56	3.74	−31.46	3.88E-03
TINAGL1	tubulointerstitial nephritis antigen-like 1	194.17	6.41	−30.30	3.88E-03
TNC	tenascin C	6.64	35.78	5.39	3.88E-03
MOCOS	molybdenum cofactor sulfurase	2.64	14.92	5.65	3.88E-03
EEF1A1P5	eukaryotic translation elongation factor 1 alpha 1 pseudogene 5	10.56	60.44	5.72	3.88E-03
DDR2	discoidin domain receptor tyrosine kinase 2	1.99	11.50	5.77	3.88E-03
STC1	stanniocalcin 1	3.78	27.66	7.32	3.88E-03
GDF15	growth differentiation factor 15	58.10	440.55	7.58	3.88E-03
IL1B	interleukin 1, beta	8.99	80.74	8.98	3.88E-03
FTH1P8	ferritin, heavy polypeptide 1 pseudogene 8	1.56	14.42	9.27	2.88E-02
FTH1P11	ferritin, heavy polypeptide 1 pseudogene 11	8.09	77.13	9.54	3.88E-03
RNA5-8SP6	RNA, 5.8S ribosomal pseudogene 6	2098.79	20220.80	9.63	1.16E-02
MME	membrane metallo-endopeptidase	3.76	36.45	9.68	3.88E-03
NAMPT	nicotinamide phosphoribosyltransferase	3.67	36.39	9.91	3.88E-03
IL1A	interleukin 1, alpha	0.69	11.02	16.04	1.37E-02
CXCL5	chemokine (C-X-C motif) ligand 5	1.45	23.55	16.22	3.88E-03
PTGS2	prostaglandin-endoperoxide synthase 2	11.30	190.10	16.83	3.88E-03
IL8	interleukin 8	32.00	678.70	21.21	3.88E-03
CXCR7	chemokine (C-X-C motif) receptor 7	0.51	10.79	21.23	3.88E-03
SLC39A8	solute carrier family 39 (zinc transporter), member 8	0.97	29.35	30.30	3.88E-03
TNFAIP6	tumor necrosis factor, alpha-induced protein 6	0.46	38.60	83.83	4.35E-02
CDR1	cerebellar degeneration-related protein 1, 34 kDa	0.29	31.82	110.58	3.88E-03

### Diverse and dominant pathways in HKCs

**Figure 1:**
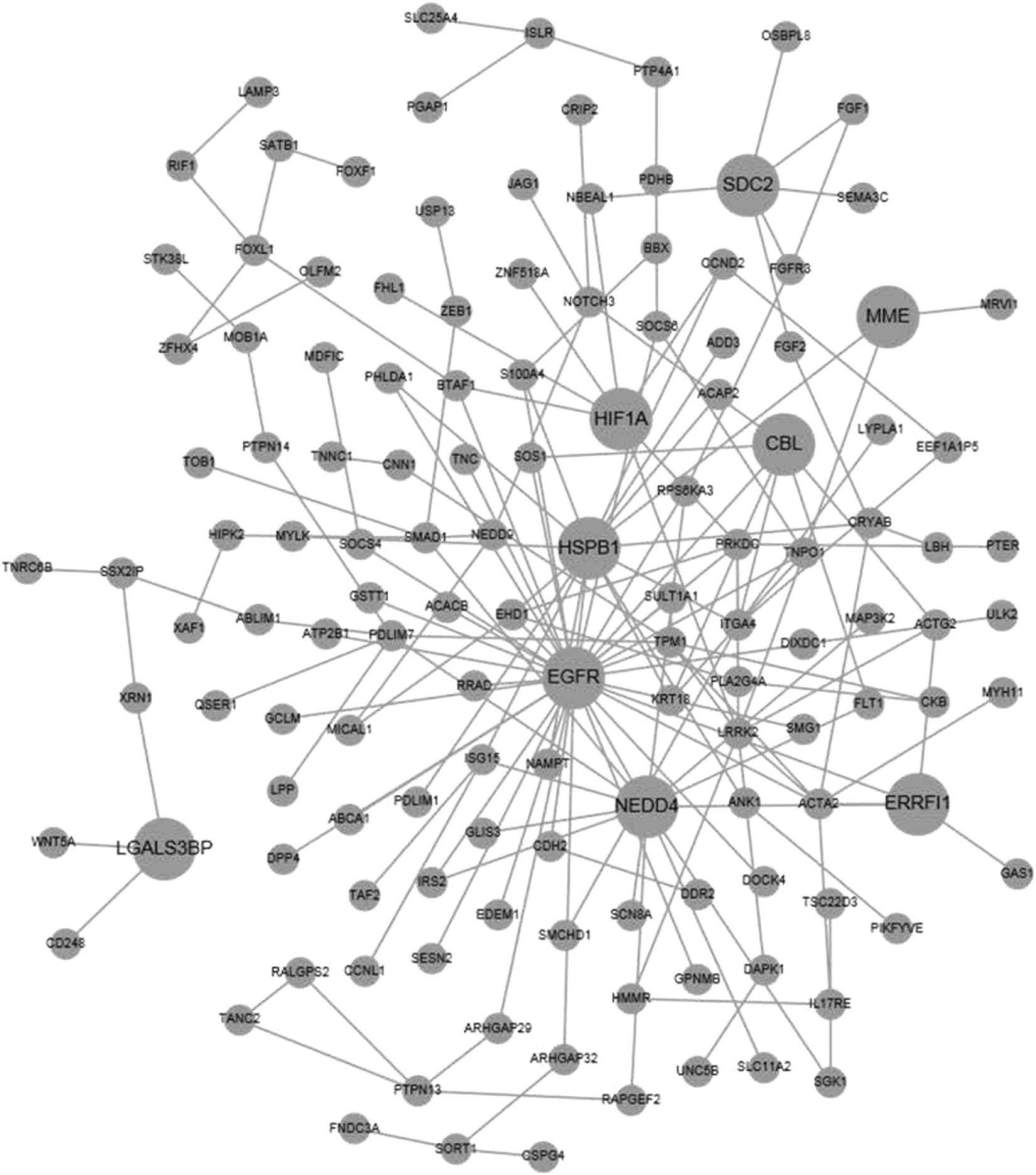
Protein-protein interaction networks derived from the differentially expressed genes in keratoconic corneal stromal cells. The larger size of the node indicates the more interactions with other genes.

Gene ontology analysis using all 423 genes indicated the significant enrichment of genes coding for proteins involved in or related with cell migration, collagen-containing ECM, adherens junction, intrinsic to plasma membrane, cytokine and growth factor activity, growth factor binding, and kinase activity. KEGG (Kyoto Encyclopedia of Genes and Genomes) pathway analysis identified the enriched pathways including MAPK signaling and Rap1 signaling. Reactome analysis indicated the involvement of PTK6 promoting HIF1A stabilization, non-integrin membrane-ECM interactions, ECM organization, and syndecan interactions. Wikipathway analysis suggested the enrichment of genes in MAPK signaling, differentiation, and focal adhesion-PI3K-Akt-mTOR-signaling pathway. Seven genes (DAPK1, ELK4, HIF1A, MEF2C, RPS6KA3, and SGK1) are targets of MAPK7 kinase activity. Many of the differentially expressed genes are enriched targets of miR-26a/b, miR-203, miR-380-3p, and miR-96 with 28, 23, 12, and 22 target genes respectively. Our miRNA profiling in seven normal human corneal tissues^39^ indicated the expression of these miRNAs, suggesting their potential role in corneal function. Protein network analysis identified a subnetwork with several connection hubs or seeds, including EGFR, NEDD4, SNTA1, LGALS3BP, HSPB1, SDC2, MME, HIF1A, CBL, and ERRFI1 (Fig. 1).

With the relatively highly expressed 208 differential genes, gene ontology analysis indicated the enrichment of genes involved with cell migration/motility, focal adhesion, endoplasmic reticulum lumen, and ECM, similar to the bioinformatics findings with all 423 genes.

### RT-PCR validation

**Figure 2:**
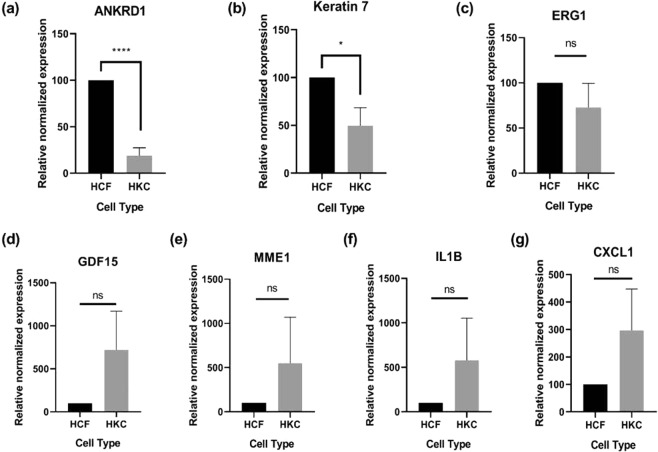
Real-time PCR validation of selected genes for their differential expression from RNA-Seq. The expression of (**a**) ANKRD1, and (**b**) Keratin7 was successfully validated using RT-PCR with significant differential expression with p values < 0.0001, and 0.0365 respectively. The expression of (**c**) ERG1, (**d**) GDF15, (**e**) MME1, (**f**) IL1B, and (**g**) CXCL1 was not validated for their differential expression from RNA-Seq (p value > 0.05).

We further validated the differential gene expression by selecting seven genes from the top 20 genes identified by transcriptomics. We focused on novel pathways associated with proliferation, wound healing, and pro-inflammatory pathways. We compared our RNA-Seq results with RNA expression using RT-PCR. Of the genes tested, ANKRD1 and KRT7 were significantly down-regulated in HKCs when compared to controls (Fig. 2), consistent with our RNA-Seq findings. In contrast, EGR1, IL1B, GDF15, MME, and CXCL1 RNA expression was not significantly different between HKCs and Controls, where RNA-Seq analysis showed significant up-regulation in HKCs (Fig. 2).

## Discussion

Previous reports applying transcript and gene analysis approaches in KC have identified altered expression ranging from a diverse group of pathways, including expression of keratocan, ECM proteins, Wnt signaling, lysyl oxidase, and inflammatory genes^12–24^. These studies have highlighted the heterogeneity of the KC condition and the need to identify common biomarkers of KC. In this study, we investigated whether gene expression in corneal stromal cells is altered by KC. For the first time, RNA-Seq was used to compare the global gene expression of the corneal stroma derived cells from KC and healthy controls. Bioinformatics were used to delineate critical pathways and highly modulated genes were validated by RT-PCR. Our analysis indicated the potential involvement of several pathways in KC pathogenesis via stromal cells, including cell migration, cellular adherence, and ECM.

The transcriptional factor, ANKRD1, is known to function in wound healing responses in cardiac tissue^40^ via regulation of the ERK and TGF-β pathways, among others^41,42^, though its expression and role in the cornea has yet to be reported. Furthermore, knockout of ANKRD1 in the mouse has shown delayed wound healing in skin tissue, due to defects in interactions between dermal fibroblasts and the surrounding ECM^43^. The interplay of ANKRD1 expression and the TGF-β pathway has also been reported^44,45^, further establishing a functional role in wound healing^46^. Interestingly, in our study, we have identified a downregulation of ANKRD1 in HKCs under homeostatic conditions validated in both the RNA-Seq and RT-PCR-data, which supports the hypothesis of an abnormal wound healing phenotype in KC^47,48^. TGF-β is known to also regulate Keratin7^49^. Keratin 7 is a protein coding gene, which plays a role in DNA synthesis. Krenzer and Freddo reported Keratin 7 expression in the conjunctiva^50^. Elder *et al*. described Keratin 7 in the basal and suprabasal epithelial cells of the central cornea^51^. However, it was not found in the central corneal epithelium^52^.

While Keratin 7 is not normally reported in the corneal stromal layer or in KC studies, our data shows downregulation of Keratin 7 in HKCs. This finding could indicate a role in the context of wound healing and loss of corneal transparency. Keratin 7 isoform polymerizes to form the intermediate cytoskeletal filaments that provide structure and stability to corneal epithelial cells. Thus, it is associated with cytoskeletal signaling and remodeling pathways^49^. Through many factors and specifically TGF-β regulation, Keratin 7 has also been implicated in cellular stress responses to wound healing and tissue repair^53,54^. Acting through the recruitment and activation of SMADs to regulate gene expression. We, and others, have reported the role of SMADs in KC^55–59^. Given that KC has previously been associated with an altered response to TGF-β1 and TGF-β3 ligands^57,60^, the downregulation of both ANKRD1 and Keratin 7 in HKCs may contribute to this complex interplay favoring a pro-fibrotic phenotype in HKCs compared to healthy controls. The functional relevance of our findings to the KC condition may be related to these downstream pathways regulated by TGF-β signaling, a key pathway important in wound healing and ECM deposition in corneal biology^61^. Further studies to determine the functional effects of decreased gene expression in the context of KC development or progression are warranted. Moreover, validation of protein level changes are needed to determine that altered gene expression identified in our study contributes to protein level changes.

Our study also has a few limitations. Firstly, the sample size is relatively small. Large sample size of both cases and controls with similar age range will definitely increase the statistical power to detect additional signaling pathways and networks. Secondly, we have used primary corneal stromal fibroblast cells instead of the isolated stromal layer tissue. The culture process may influence the expression profile. However, studies from our lab^27,57^ and others^62,63^ have consistently supported maintenance of an altered disease phenotype by KC-derived stromal cells cultured *in vitro* with characteristics parallel to the *in vivo* condition, including altered ECM expression and regulation^9^. Thirdly, the identified KC-associated signaling pathways and networks could either be the disease outcome or may actively contribute to KC pathogenesis, which is difficult to discern in a snapshot of transcript levels. A systematic approach to determine the role of specific pathways in KC development from onset to further progression to the late-stage disease is required to validate disease causation. However, this is difficult with the lack of tissue/cell isolation from early-stage KC patients. Lastly, the age differences between control and KC patients was ~13.6 years with an average age of 47.2 ± 18.3 years for controls and 60.8 ± 11.6 years for KC. The effects of age on gene expression has previously been reported with downregulation associated with collagen^64^ and elastin expression by fibroblasts of the skin^65^. Likewise, aging influences corneal structure with increased keratocyte senescence and altered ECM rigidity^66^. Thus, the age gap in our study between groups may have contributed to differential gene expression independent of the KC disease.

In summary, using RNA-Seq technology, we have successfully profiled the differential gene expression in primary human corneal stromal cells derived from patients with KC. This data will advance our molecular understanding of the pathogenesis of KC.

## Supplementary information

Supplementary

## Data Availability

The datasets generated during the current study are available from the corresponding author on reasonable request.
