# Supplementary material for: Transcriptional profiling of corneal stromal cells derived from patients with keratoconus
Source: Sci Rep. 2019 Aug 29;9:12567. doi: 10.1038/s41598-019-48983-8 (PMC6715750; doi:10.1038/s41598-019-48983-8)
Supplement: Supplementary file 1 — Supplementary [file 41598_2019_48983_MOESM1_ESM.docx]

**Transcriptional profiling of corneal stromal cells derived from patients with keratoconus**

*Sharif Rabab^1$^, Mariam L. Khaled^2$^, Tina B. McKay^3^, Yutao Liu^2*^, Dimitrios Karamichos^1,4*^*

*^1^* Department of Cell Biology, University of Oklahoma Health science Center, Oklahoma City, Oklahoma, 73104, USA

*^2^* Cellular Biology & Anatomy, Augusta University, Augusta, GA 30912, United States.

*^3^* Schepens Eye Research Institute and Department of Ophthalmology, Harvard Medical School, Boston, MA, 02114, USA

*^4^* Department of Ophthalmology/Dean McGee Eye Institute, University of Oklahoma Health Science Center, Oklahoma City, Oklahoma, 73104, USA

**^$^ Both authors contributed equally to this work.**

***Co-Corresponding authors:** Dimitrios Karamichos and Yutao Liu: [Dimitrios-Karamichos@ouhsc.edu](mailto:Dimitrios-Karamichos@ouhsc.edu) / [yutliu@augusta.edu](mailto:YUTLIU@augusta.edu)

Supplemental Table 1. List of differentially expressed coding RNAs in Keratoconus-derived human corneal fibroblast vs. normal controls.

| **Gene name** | **Gene Description** | **Locus** | **Normalized Average expression** | | **Fold change** | **FDR value** |
| --- | --- | --- | --- | --- | --- | --- |
|  |  |  | **Controls (n=5)** | **KC (n=4)** |  |  |
| ESAM | endothelial cell adhesion molecule | 11:124609828-124635832 | 5.30 | 0.01 | -482.31 | 3.88E-03 |
| ANKRD1 | ankyrin repeat domain 1 (cardiac muscle) | 10:92671852-92681033 | 119.62 | 0.28 | -428.03 | 3.88E-03 |
| AQP1 | aquaporin 1 (Colton blood group) | 7:30692199-30965131 | 17.25 | 0.12 | -145.47 | 3.88E-03 |
| MYH11 | myosin, heavy chain 11, smooth muscle | 16:15737123-15950890 | 17.91 | 0.14 | -130.62 | 4.16E-02 |
| MRVI1 | murine retrovirus integration site 1 homolog | 11:10562818-10715535 | 17.32 | 0.15 | -117.10 | 3.88E-03 |
| CRYAB | crystallin, alpha B | 11:111779288-111797596 | 1632.04 | 14.65 | -111.37 | 3.88E-03 |
| A2M | alpha-2-macroglobulin | 12:9217772-9268825 | 17.67 | 0.16 | -108.64 | 3.88E-03 |
| OXTR | oxytocin receptor | 3:8661085-9005457 | 52.23 | 0.65 | -80.61 | 9.23E-03 |
| ANO1 | anoctamin 1, calcium activated chloride channel | 11:69924407-70035634 | 4.58 | 0.06 | -73.98 | 3.20E-02 |
| ACTA2 | actin, alpha 2, smooth muscle, aorta | 10:90639490-90775542 | 4845.11 | 65.95 | -73.47 | 3.88E-03 |
| SCUBE3 | signal peptide, CUB domain, EGF-like 3 | 6:35182189-35220856 | 10.72 | 0.15 | -69.63 | 3.88E-03 |
| LGI4 | leucine-rich repeat LGI family, member 4 | 19:35615416-35645204 | 13.20 | 0.25 | -53.71 | 1.37E-02 |
| PDLIM3 | PDZ and LIM domain 3 | 4:186422902-186456766 | 20.99 | 0.46 | -45.58 | 3.88E-03 |
| ITGA7 | integrin, alpha 7 | 12:56075329-56118489 | 91.90 | 2.11 | -43.46 | 3.47E-02 |
| MCAM | melanoma cell adhesion molecule | 11:119179240-119191799 | 75.51 | 1.74 | -43.32 | 3.88E-03 |
| COL5A3 | collagen, type V, alpha 3 | 19:10070236-10121147 | 16.03 | 0.40 | -40.38 | 3.88E-03 |
| GALNTL4 | UDP-N-acetyl-alpha-D-galactosamine:polypeptide N-acetylgalactosaminyltransferase-like 4 | 11:11292422-11643552 | 9.61 | 0.24 | -39.73 | 6.70E-03 |
| RGS5 | regulator of G-protein signaling 5 | 1:163080910-163325554 | 10.16 | 0.26 | -39.21 | 9.23E-03 |
| ID4 | inhibitor of DNA binding 4, dominant negative helix-loop-helix protein | 6:19837616-19840915 | 34.13 | 0.89 | -38.35 | 3.88E-03 |
| LMCD1 | LIM and cysteine-rich domains 1 | 3:7994491-8653610 | 36.38 | 1.00 | -36.34 | 3.88E-03 |
| NRXN2 | neurexin 2 | 11:64373645-64490660 | 5.76 | 0.17 | -34.49 | 3.88E-03 |
| SGCA | sarcoglycan, alpha (50kDa dystrophin-associated glycoprotein) | 17:48240883-48258539 | 34.86 | 1.03 | -33.81 | 3.88E-03 |
| HSPB7 | heat shock 27kDa protein family, member 7 (cardiovascular) | 1:16340522-16360545 | 117.56 | 3.74 | -31.46 | 3.88E-03 |
| TINAGL1 | tubulointerstitial nephritis antigen-like 1 | 1:32042115-32053288 | 194.17 | 6.41 | -30.30 | 3.88E-03 |
| JAG1 | jagged 1 | 20:10618331-10654608 | 17.85 | 0.62 | -28.55 | 3.88E-03 |
| HAPLN3 | hyaluronan and proteoglycan link protein 3 | 15:89420518-89438857 | 52.94 | 2.10 | -25.26 | 3.88E-03 |
| KRT7 | keratin 7 | 12:52626303-52715182 | 1280.39 | 50.74 | -25.24 | 3.88E-03 |
| ADAMTS15 | ADAM metallopeptidase with thrombospondin type 1 motif, 15 | 11:130318868-130346532 | 3.46 | 0.15 | -22.41 | 3.88E-03 |
| CCND2 | cyclin D2 | 12:4357930-4414516 | 1.76 | 0.08 | -21.55 | 3.88E-03 |
| ISLR | immunoglobulin superfamily containing leucine-rich repeat | 15:74445140-74471567 | 18.24 | 0.87 | -21.05 | 2.04E-02 |
| DAPK1 | death-associated protein kinase 1 | 9:90112142-90323548 | 1.84 | 0.09 | -20.92 | 3.88E-03 |
| NREP | neuronal regeneration related protein homolog (rat) | 5:110831730-111353006 | 148.69 | 7.45 | -19.96 | 6.70E-03 |
| WFDC1 | WAP four-disulfide core domain 1 | 16:84328251-84363450 | 277.41 | 13.92 | -19.92 | 3.88E-03 |
| KRT18 | keratin 18 | 12:53290970-53346686 | 319.77 | 16.31 | -19.60 | 3.88E-03 |
| MGP | matrix Gla protein | 12:14956505-15059520 | 163.02 | 8.35 | -19.52 | 3.88E-03 |
| FAM212B | family with sequence similarity 212, member B | 1:112025969-112310638 | 7.06 | 0.38 | -18.80 | 4.72E-02 |
| LBH | limb bud and heart development homolog (mouse) | 2:30454396-30546596 | 7.42 | 0.41 | -18.07 | 3.20E-02 |
| CMKLR1 | chemokine-like receptor 1 | 12:108681820-108733118 | 4.38 | 0.24 | -17.86 | 3.88E-03 |
| GALNTL1 | UDP-N-acetyl-alpha-D-galactosamine:polypeptide N-acetylgalactosaminyltransferase-like 1 | 14:69649736-69821183 | 1.32 | 0.07 | -17.69 | 9.23E-03 |
| PTN | pleiotrophin | 7:136912087-137028611 | 9.45 | 0.55 | -17.13 | 4.35E-02 |
| RASGRP3 | RAS guanyl releasing protein 3 (calcium and DAG-regulated) | 2:33661390-33789817 | 2.00 | 0.12 | -16.97 | 3.88E-03 |
| C21orf7 | chromosome 21 open reading frame 7 | 21:30449791-30548210 | 15.33 | 0.90 | -16.95 | 3.88E-03 |
| LMOD1 | leiomodin 1 (smooth muscle) | 1:201862969-201915715 | 28.81 | 1.72 | -16.77 | 3.88E-03 |
| NR2F2-AS1 | NR2F2 antisense RNA 1 | 15:96670597-96883492 | 1.53 | 0.09 | -16.29 | 1.61E-02 |
| LINC00672 | long intergenic non-protein coding RNA 672 | 17:37081420-37084310 | 1.94 | 0.13 | -15.07 | 1.16E-02 |
| HES4 | hairy and enhancer of split 4 (Drosophila) | 1:934341-935552 | 126.99 | 8.91 | -14.25 | 3.88E-03 |
| C1orf167 | chromosome 1 open reading frame 167 | 1:11821843-11908402 | 0.71 | 0.05 | -14.10 | 4.35E-02 |
| OLFML2B | olfactomedin-like 2B | 1:161952981-161993644 | 39.76 | 2.89 | -13.74 | 3.88E-03 |
| FAM46B | family with sequence similarity 46, member B | 1:27331510-27339327 | 6.77 | 0.50 | -13.67 | 3.88E-03 |
| TNNC1 | troponin C type 1 (slow) | 3:52485117-52488086 | 17.96 | 1.33 | -13.51 | 3.88E-03 |
| TMC6 | transmembrane channel-like 6 | 17:76000248-76139049 | 5.86 | 0.44 | -13.43 | 6.70E-03 |
| PRELP | proline/arginine-rich end leucine-rich repeat protein | 1:203444955-203460480 | 8.14 | 0.61 | -13.31 | 3.88E-03 |
| PALM | paralemmin | 19:708952-748329 | 18.65 | 1.41 | -13.18 | 3.88E-03 |
| CSPG4 | chondroitin sulfate proteoglycan 4 | 15:75966662-76005189 | 22.62 | 1.74 | -12.97 | 3.88E-03 |
| OLFM2 | olfactomedin 2 | 19:9964393-10047228 | 40.11 | 3.24 | -12.39 | 3.88E-03 |
| LIMS2 | LIM and senescent cell antigen-like domains 2 | 2:128395955-128439360 | 189.90 | 16.06 | -11.83 | 3.88E-03 |
| COL3A1 | collagen, type III, alpha 1 | 2:189839045-189877472 | 297.87 | 25.27 | -11.79 | 3.88E-03 |
| SORT1 | sortilin 1 | 1:109852191-109940573 | 9.59 | 0.83 | -11.56 | 3.88E-03 |
| SYNPO2 | synaptopodin 2 | 4:119809995-119982402 | 15.10 | 1.31 | -11.49 | 3.88E-03 |
| DAAM2 | dishevelled associated activator of morphogenesis 2 | 6:39760141-39902290 | 5.66 | 0.49 | -11.48 | 6.70E-03 |
| S100A4 | S100 calcium binding protein A4 | 1:153516088-153522612 | 3694.78 | 330.84 | -11.17 | 3.88E-03 |
| MEF2C | myocyte enhancer factor 2C | 5:87803362-88762215 | 0.70 | 0.07 | -10.55 | 3.88E-03 |
| NOTCH3 | notch 3 | 19:15270443-15311792 | 47.81 | 4.59 | -10.41 | 3.88E-03 |
| CELSR2 | cadherin, EGF LAG seven-pass G-type receptor 2 (flamingo homolog, Drosophila) | 1:109792640-109818377 | 1.72 | 0.17 | -10.17 | 3.20E-02 |
| IL34 | interleukin 34 | 16:70613797-70694585 | 16.33 | 1.61 | -10.15 | 6.70E-03 |
| TSPAN2 | tetraspanin 2 | 1:115590631-115632121 | 1.68 | 0.17 | -10.12 | 3.88E-03 |
| CNN1 | calponin 1, basic, smooth muscle | 19:11649531-11661138 | 213.75 | 21.12 | -10.12 | 3.88E-03 |
| CABP1 | calcium binding protein 1 | 12:121067907-121105127 | 4.49 | 0.48 | -9.41 | 4.03E-02 |
| BAMBI | BMP and activin membrane-bound inhibitor homolog (Xenopus laevis) | 10:28966270-28971868 | 21.42 | 2.31 | -9.29 | 3.88E-03 |
| MPP7 | membrane protein, palmitoylated 7 (MAGUK p55 subfamily member 7) | 10:28339921-28591995 | 0.42 | 0.05 | -9.26 | 1.37E-02 |
| CKB | creatine kinase, brain | 14:103985995-103989448 | 105.09 | 11.75 | -8.94 | 3.88E-03 |
| PDGFA | platelet-derived growth factor alpha polypeptide | 7:536894-559933 | 35.58 | 4.38 | -8.13 | 3.88E-03 |
| FHL1 | four and a half LIM domains 1 | X:135229558-135293518 | 207.80 | 25.69 | -8.09 | 3.88E-03 |
| FGF1 | fibroblast growth factor 1 (acidic) | 5:141689991-142077617 | 24.75 | 3.06 | -8.08 | 3.88E-03 |
| INHBA | inhibin, beta A | 7:41706765-41818986 | 9.84 | 1.22 | -8.05 | 3.88E-03 |
| PLAC9 | placenta-specific 9 | 10:81891476-81905115 | 151.76 | 19.52 | -7.78 | 3.88E-03 |
| CDK18 | cyclin-dependent kinase 18 | 1:205473722-205501921 | 10.06 | 1.30 | -7.72 | 3.88E-03 |
| RRAD | Ras-related associated with diabetes | 16:66955581-66959547 | 50.58 | 6.78 | -7.46 | 3.88E-03 |
| CASZ1 | castor zinc finger 1 | 1:10696660-10856707 | 0.65 | 0.09 | -7.35 | 2.04E-02 |
| IGFBP7 | insulin-like growth factor binding protein 7 | 4:57829535-58071676 | 1256.86 | 171.41 | -7.33 | 6.70E-03 |
| RASL11A | RAS-like, family 11, member A | 13:27844463-27847827 | 33.82 | 4.72 | -7.17 | 3.88E-03 |
| LFNG | LFNG O-fucosylpeptide 3-beta-N-acetylglucosaminyltransferase | 7:2552162-2568811 | 3.06 | 0.44 | -6.92 | 6.70E-03 |
| FLT1 | fms-related tyrosine kinase 1 (vascular endothelial growth factor/vascular permeability factor receptor) | 13:28874488-29069265 | 6.54 | 0.97 | -6.76 | 3.88E-03 |
| ANK1 | ankyrin 1, erythrocytic | 8:41510738-41754280 | 1.96 | 0.29 | -6.68 | 3.88E-03 |
| TPPP3 | tubulin polymerization-promoting protein family member 3 | 16:67423711-67427438 | 20.09 | 3.02 | -6.66 | 3.88E-03 |
| PPFIBP2 | PTPRF interacting protein, binding protein 2 (liprin beta 2) | 11:7534528-7678358 | 33.10 | 5.07 | -6.53 | 1.37E-02 |
| FAM43A | family with sequence similarity 43, member A | 3:194406621-194409762 | 11.72 | 1.81 | -6.49 | 3.88E-03 |
| CORIN | corin, serine peptidase | 4:47596014-47840123 | 8.76 | 1.37 | -6.41 | 2.24E-02 |
| LRRC32 | leucine rich repeat containing 32 | 11:76368099-76381791 | 58.64 | 9.19 | -6.38 | 2.04E-02 |
| TRIM47 | tripartite motif containing 47 | 17:73870241-73875627 | 34.37 | 5.52 | -6.23 | 9.23E-03 |
| COX7A1 | cytochrome c oxidase subunit VIIa polypeptide 1 (muscle) | 19:36641823-36643771 | 257.59 | 42.22 | -6.10 | 3.88E-03 |
| SYTL2 | synaptotagmin-like 2 | 11:85405266-85522184 | 2.58 | 0.43 | -6.02 | 3.88E-03 |
| ACTG2 | actin, gamma 2, smooth muscle, enteric | 2:74119440-74146992 | 390.54 | 67.36 | -5.80 | 4.16E-02 |
| DACT1 | dapper, antagonist of beta-catenin, homolog 1 (Xenopus laevis) | 14:59100684-59115039 | 5.04 | 0.89 | -5.70 | 3.88E-03 |
| TCF7L1 | transcription factor 7-like 1 (T-cell specific, HMG-box) | 2:85360532-85537511 | 17.08 | 3.04 | -5.62 | 3.88E-03 |
| KALRN | kalirin, RhoGEF kinase | 3:123798869-124440036 | 0.48 | 0.09 | -5.54 | 3.88E-03 |
| C7orf10 | chromosome 7 open reading frame 10 | 7:40174574-40900362 | 72.13 | 13.09 | -5.51 | 3.47E-02 |
| BMP4 | bone morphogenetic protein 4 | 14:54416453-54425479 | 2.11 | 0.38 | -5.51 | 4.27E-02 |
| GADD45B | growth arrest and DNA-damage-inducible, beta | 19:2476119-2478257 | 77.20 | 14.64 | -5.27 | 3.88E-03 |
| CSRP2 | cysteine and glycine-rich protein 2 | 12:77252002-77274132 | 116.63 | 22.55 | -5.17 | 3.88E-03 |
| HYAL1 | hyaluronoglucosaminidase 1 | 3:50337319-50349812 | 1.13 | 0.22 | -5.16 | 1.16E-02 |
| SUSD2 | sushi domain containing 2 | 22:24577226-24585078 | 58.18 | 11.41 | -5.10 | 3.88E-03 |
| MFGE8 | milk fat globule-EGF factor 8 protein | 15:89441915-89456642 | 744.13 | 146.76 | -5.07 | 1.83E-02 |
| LZTS1 | leucine zipper, putative tumor suppressor 1 | 8:20103675-20161474 | 1.60 | 0.32 | -5.06 | 1.16E-02 |
| TPM1 | tropomyosin 1 (alpha) | 15:63334830-63364114 | 458.04 | 91.41 | -5.01 | 1.16E-02 |
| KHDRBS3 | KH domain containing, RNA binding, signal transduction associated 3 | 8:136469699-136668965 | 5.01 | 1.02 | -4.89 | 6.70E-03 |
| CXXC5 | CXXC finger protein 5 | 5:139026883-139063467 | 37.46 | 7.80 | -4.80 | 3.88E-03 |
| SOX13 | SRY (sex determining region Y)-box 13 | 1:204042242-204096863 | 1.75 | 0.37 | -4.74 | 2.56E-02 |
| TRPV2 | transient receptor potential cation channel, subfamily V, member 2 | 17:16317837-16340317 | 80.31 | 17.36 | -4.63 | 3.88E-03 |
| FGFR3 | fibroblast growth factor receptor 3 | 4:1795033-1810599 | 0.58 | 0.13 | -4.59 | 2.88E-02 |
| STK38L | serine/threonine kinase 38 like | 12:27396900-27478892 | 4.86 | 1.07 | -4.56 | 3.88E-03 |
| OSR1 | odd-skipped related 1 (Drosophila) | 2:19551245-19558414 | 8.39 | 1.85 | -4.53 | 1.37E-02 |
| IL17RE | interleukin 17 receptor E | 3:9944295-9958086 | 3.16 | 0.70 | -4.48 | 4.03E-02 |
| HERC6 | HECT and RLD domain containing E3 ubiquitin protein ligase family member 6 | 4:89299890-89364263 | 2.47 | 0.56 | -4.42 | 3.88E-03 |
| COL5A1 | collagen, type V, alpha 1 | 9:137533619-137736686 | 117.63 | 26.97 | -4.36 | 3.88E-03 |
| DSP | desmoplakin | 6:7541807-7586950 | 6.97 | 1.60 | -4.36 | 3.88E-03 |
| ISG15 | ISG15 ubiquitin-like modifier | 1:948802-949920 | 519.58 | 119.30 | -4.36 | 3.88E-03 |
| TP53I11 | tumor protein p53 inducible protein 11 | 11:44740941-44972840 | 61.72 | 14.48 | -4.26 | 3.88E-03 |
| HSPB1 | heat shock 27kDa protein 1 | 7:75931860-75933612 | 3288.73 | 780.07 | -4.22 | 9.23E-03 |
| BGN | biglycan | X:152710177-152775012 | 448.03 | 108.32 | -4.14 | 1.83E-02 |
| F10 | coagulation factor X | 13:113777127-113803843 | 25.83 | 6.27 | -4.12 | 3.88E-03 |
| PRRX2 | paired related homeobox 2 | 9:132427919-132484875 | 370.94 | 90.00 | -4.12 | 3.88E-03 |
| HRCT1 | histidine rich carboxyl terminus 1 | 9:35906188-35907138 | 14.97 | 3.68 | -4.07 | 3.47E-02 |
| MYLK | myosin light chain kinase | 3:123304388-123603178 | 5.88 | 1.45 | -4.07 | 3.88E-03 |
| EFNB3 | ephrin-B3 | 17:7608519-7614696 | 2.33 | 0.57 | -4.07 | 3.20E-02 |
| SGK223 | homolog of rat pragma of Rnd2 | 8:8175257-8244008 | 3.22 | 0.79 | -4.07 | 3.78E-02 |
| TPD52L1 | tumor protein D52-like 1 | 6:125440194-125585553 | 41.00 | 10.11 | -4.06 | 3.88E-03 |
| SHROOM3 | shroom family member 3 | 4:77356252-77723117 | 2.05 | 0.51 | -4.05 | 3.88E-03 |
| PLAU | plasminogen activator, urokinase | 10:75668934-75682535 | 42.81 | 10.84 | -3.95 | 3.88E-03 |
| BCAM | basal cell adhesion molecule (Lutheran blood group) | 19:45312327-45324673 | 85.20 | 21.85 | -3.90 | 3.88E-03 |
| SLC1A4 | solute carrier family 1 (glutamate/neutral amino acid transporter), member 4 | 2:65215610-65250999 | 23.27 | 6.11 | -3.81 | 3.88E-03 |
| EHD1 | EH-domain containing 1 | 11:64619113-64655768 | 112.81 | 29.67 | -3.80 | 3.88E-03 |
| SEMA3B | sema domain, immunoglobulin domain (Ig), short basic domain, secreted, (semaphorin) 3B | 3:50304989-50314977 | 23.07 | 6.11 | -3.78 | 9.23E-03 |
| HTRA1 | HtrA serine peptidase 1 | 10:124221040-124274424 | 704.75 | 189.71 | -3.71 | 4.35E-02 |
| MOCS1 | molybdenum cofactor synthesis 1 | 6:39760141-39902290 | 13.04 | 3.56 | -3.66 | 1.83E-02 |
| SPON2 | spondin 2, extracellular matrix protein | 4:1160719-1202750 | 49.30 | 13.71 | -3.59 | 3.88E-03 |
| SNTA1 | syntrophin, alpha 1 | 20:31995760-32031698 | 55.75 | 15.55 | -3.59 | 3.88E-03 |
| NR2F2 | nuclear receptor subfamily 2, group F, member 2 | 15:96670597-96883492 | 9.92 | 2.77 | -3.58 | 3.88E-03 |
| NLRC5 | NLR family, CARD domain containing 5 | 16:57023396-57117443 | 8.27 | 2.33 | -3.55 | 2.88E-02 |
| SULT1A1 | sulfotransferase family, cytosolic, 1A, phenol-preferring, member 1 | 16:28616902-28634946 | 10.67 | 3.02 | -3.53 | 4.03E-02 |
| LGALS3BP | lectin, galactoside-binding, soluble, 3 binding protein | 17:76967319-76976191 | 230.76 | 65.53 | -3.52 | 3.88E-03 |
| C6orf132 | chromosome 6 open reading frame 132 | 6:42068855-42110357 | 3.69 | 1.05 | -3.52 | 4.72E-02 |
| FBLN2 | fibulin 2 | 3:13573823-13679922 | 26.39 | 7.53 | -3.50 | 4.35E-02 |
| ABLIM1 | actin binding LIM protein 1 | 10:116190871-116444414 | 0.89 | 0.26 | -3.48 | 1.37E-02 |
| ERRFI1 | ERBB receptor feedback inhibitor 1 | 1:8064463-8086368 | 42.37 | 12.22 | -3.47 | 3.88E-03 |
| CALHM2 | calcium homeostasis modulator 2 | 10:105206542-105222452 | 20.92 | 6.06 | -3.45 | 3.88E-03 |
| SDC2 | syndecan 2 | 8:97505578-97624000 | 99.60 | 29.09 | -3.42 | 1.16E-02 |
| RHOJ | ras homolog family member J | 14:63670831-63759937 | 6.05 | 1.77 | -3.42 | 3.88E-03 |
| PLEKHG3 | pleckstrin homology domain containing, family G (with RhoGef domain) member 3 | 14:65170819-65346601 | 4.18 | 1.24 | -3.37 | 3.37E-02 |
| NLRP1 | NLR family, pyrin domain containing 1 | 17:5402746-5522744 | 97.89 | 29.37 | -3.33 | 3.37E-02 |
| A4GALT | alpha 1,4-galactosyltransferase | 22:43088126-43117304 | 27.50 | 8.28 | -3.32 | 6.70E-03 |
| PTPLA | protein tyrosine phosphatase-like (proline instead of catalytic arginine), member A | 10:17631957-17659376 | 42.32 | 13.13 | -3.22 | 2.88E-02 |
| TPST2 | tyrosylprotein sulfotransferase 2 | 22:26921457-26992681 | 52.16 | 16.20 | -3.22 | 3.88E-03 |
| NEDD9 | neural precursor cell expressed, developmentally down-regulated 9 | 6:11173684-11382581 | 6.51 | 2.06 | -3.16 | 6.70E-03 |
| DIXDC1 | DIX domain containing 1 | 11:111797867-111893308 | 2.57 | 0.83 | -3.11 | 2.04E-02 |
| CD97 | CD97 molecule | 19:14491312-14519537 | 56.87 | 18.39 | -3.09 | 3.88E-03 |
| CRIP2 | cysteine-rich protein 2 | 14:105939298-105946499 | 553.88 | 179.88 | -3.08 | 1.37E-02 |
| STC2 | stanniocalcin 2 | 5:172741715-172756506 | 74.33 | 24.45 | -3.04 | 3.78E-02 |
| PDLIM1 | PDZ and LIM domain 1 | 10:96997328-97050781 | 153.04 | 50.77 | -3.01 | 3.88E-03 |
| RTN2 | reticulon 2 | 19:45988546-46005768 | 30.70 | 10.30 | -2.98 | 3.47E-02 |
| MICAL1 | microtubule associated monoxygenase, calponin and LIM domain containing 1 | 6:109765264-109804440 | 57.99 | 19.88 | -2.92 | 3.37E-02 |
| TSC22D3 | TSC22 domain family, member 3 | X:106956450-107020572 | 23.01 | 7.98 | -2.89 | 6.70E-03 |
| LOXL4 | lysyl oxidase-like 4 | 10:100007446-100028007 | 8.11 | 2.85 | -2.85 | 4.62E-02 |
| FAM69A | family with sequence similarity 69, member A | 1:93307723-93427057 | 4.16 | 1.49 | -2.79 | 4.27E-02 |
| GSTT1 | glutathione S-transferase theta 1 | 22:24376132-24384680 | 102.26 | 36.67 | -2.79 | 6.70E-03 |
| ATOH8 | atonal homolog 8 (Drosophila) | 2:85978466-86015189 | 48.95 | 17.68 | -2.77 | 3.88E-03 |
| SNAI2 | snail homolog 2 (Drosophila) | 8:49830248-49834299 | 14.28 | 5.18 | -2.76 | 1.61E-02 |
| ITGA3 | integrin, alpha 3 (antigen CD49C, alpha 3 subunit of VLA-3 receptor) | 17:48133331-48167845 | 49.61 | 18.04 | -2.75 | 4.03E-02 |
| FOXL1 | forkhead box L1 | 16:86609973-86615303 | 5.52 | 2.01 | -2.74 | 4.95E-02 |
| TMEM160 | transmembrane protein 160 | 19:47549164-47551888 | 97.12 | 35.45 | -2.74 | 3.47E-02 |
| SLC25A4 | solute carrier family 25 (mitochondrial carrier; adenine nucleotide translocator), member 4 | 4:186064394-186068434 | 55.76 | 20.62 | -2.70 | 1.83E-02 |
| ID3 | inhibitor of DNA binding 3, dominant negative helix-loop-helix protein | 1:23884408-23886285 | 518.02 | 196.76 | -2.63 | 2.40E-02 |
| NID2 | nidogen 2 (osteonidogen) | 14:52471520-52535712 | 24.34 | 9.24 | -2.63 | 2.56E-02 |
| TRIB2 | tribbles homolog 2 (Drosophila) | 2:12857014-12882860 | 9.58 | 3.65 | -2.62 | 3.91E-02 |
| BMP1 | bone morphogenetic protein 1 | 8:22022248-22069839 | 27.93 | 10.71 | -2.61 | 2.40E-02 |
| CD248 | CD248 molecule, endosialin | 11:66080323-66086708 | 208.19 | 80.67 | -2.58 | 3.88E-03 |
| TCEAL3 | transcription elongation factor A (SII)-like 3 | X:102862378-102885881 | 90.42 | 35.73 | -2.53 | 4.35E-02 |
| XAF1 | XIAP associated factor 1 | 17:6658765-6678966 | 3.75 | 1.49 | -2.53 | 9.23E-03 |
| KCNK6 | potassium channel, subfamily K, member 6 | 19:38810483-38819660 | 13.68 | 5.48 | -2.50 | 3.78E-02 |
| PLCB4 | phospholipase C, beta 4 | 20:9049409-9461889 | 3.22 | 1.31 | -2.45 | 3.64E-02 |
| XXYLT1 | xyloside xylosyltransferase 1 | 3:194789007-194991896 | 7.65 | 3.12 | -2.45 | 4.35E-02 |
| COL4A5 | collagen, type IV, alpha 5 | X:107683073-107940775 | 1.90 | 0.78 | -2.43 | 3.91E-02 |
| PDLIM7 | PDZ and LIM domain 7 (enigma) | 5:176910394-176938275 | 332.60 | 139.62 | -2.38 | 3.64E-02 |
| KLHDC3 | kelch domain containing 3 | 6:42981950-42989036 | 54.84 | 23.08 | -2.38 | 4.35E-02 |
| PDHB | pyruvate dehydrogenase (lipoamide) beta | 3:58413356-58419584 | 55.16 | 25.01 | -2.21 | 4.16E-02 |
| BOK | BCL2-related ovarian killer | 2:242483817-242513546 | 65.43 | 30.06 | -2.18 | 4.03E-02 |
| PRKCDBP | protein kinase C, delta binding protein | 11:6340175-6341877 | 359.54 | 165.43 | -2.17 | 4.72E-02 |
| MOB1A | MOB kinase activator 1A | 2:74382164-74406025 | 5.60 | 12.34 | 2.20 | 4.62E-02 |
| NSMAF | neutral sphingomyelinase (N-SMase) activation associated factor | 8:59496062-59572403 | 4.09 | 9.15 | 2.24 | 2.04E-02 |
| EGFR | epidermal growth factor receptor | 7:55086713-55324313 | 2.77 | 6.25 | 2.26 | 3.78E-02 |
| EDEM1 | ER degradation enhancer, mannosidase alpha-like 1 | 3:5229226-5261642 | 4.83 | 10.96 | 2.27 | 4.62E-02 |
| CCNL1 | cyclin L1 | 3:156864296-156878549 | 8.68 | 19.95 | 2.30 | 2.72E-02 |
| PRKDC | protein kinase, DNA-activated, catalytic polypeptide | 8:48685668-48872743 | 2.01 | 4.67 | 2.33 | 2.40E-02 |
| GPNMB | glycoprotein (transmembrane) nmb | 7:23275585-23314727 | 9.09 | 21.21 | 2.33 | 4.86E-02 |
| ELK4 | ELK4, ETS-domain protein (SRF accessory protein 1) | 1:205577070-205601090 | 0.92 | 2.16 | 2.34 | 4.95E-02 |
| LPAR1 | lysophosphatidic acid receptor 1 | 9:113635542-113800981 | 31.65 | 74.54 | 2.35 | 2.72E-02 |
| PHLDA1 | pleckstrin homology-like domain, family A, member 1 | 12:76419226-76427712 | 15.12 | 35.83 | 2.37 | 3.47E-02 |
| MLLT4 | myeloid/lymphoid or mixed-lineage leukemia (trithorax homolog, Drosophila); translocated to, 4 | 6:168227601-168372703 | 1.14 | 2.72 | 2.38 | 2.24E-02 |
| SOS1 | son of sevenless homolog 1 (Drosophila) | 2:39208536-39351486 | 0.91 | 2.18 | 2.39 | 4.72E-02 |
| KCNK2 | potassium channel, subfamily K, member 2 | 1:215179117-215410436 | 8.96 | 21.46 | 2.39 | 3.91E-02 |
| TRPM7 | transient receptor potential cation channel, subfamily M, member 7 | 15:50844669-50979012 | 1.17 | 2.81 | 2.40 | 2.40E-02 |
| PTPN13 | protein tyrosine phosphatase, non-receptor type 13 (APO-1/CD95 (Fas)-associated phosphatase) | 4:87515467-87736324 | 1.13 | 2.74 | 2.43 | 4.72E-02 |
| UHMK1 | U2AF homology motif (UHM) kinase 1 | 1:162467040-162499419 | 3.63 | 8.90 | 2.45 | 3.03E-02 |
| SIRPA | signal-regulatory protein alpha | 20:1875153-1920543 | 17.25 | 42.35 | 2.46 | 4.86E-02 |
| EFR3A | EFR3 homolog A (S. cerevisiae) | 8:132916334-133025889 | 2.38 | 5.86 | 2.46 | 3.03E-02 |
| PHF3 | PHD finger protein 3 | 6:64345724-66417118 | 1.11 | 2.74 | 2.46 | 3.03E-02 |
| LYPLA1 | lysophospholipase I | 8:54958937-55014577 | 5.29 | 13.08 | 2.47 | 3.47E-02 |
| MBNL2 | muscleblind-like splicing regulator 2 | 13:97873687-98046374 | 8.62 | 21.40 | 2.48 | 1.16E-02 |
| XRN1 | 5'-3' exoribonuclease 1 | 3:142025448-142166904 | 0.79 | 1.96 | 2.49 | 1.61E-02 |
| SMCHD1 | structural maintenance of chromosomes flexible hinge domain containing 1 | 18:2655736-2833063 | 0.73 | 1.83 | 2.49 | 1.83E-02 |
| BBX | bobby sox homolog (Drosophila) | 3:107241782-107530171 | 1.77 | 4.43 | 2.50 | 4.27E-02 |
| BMPER | BMP binding endothelial regulator | 7:33944522-34195484 | 8.58 | 21.48 | 2.50 | 3.91E-02 |
| SECISBP2L | SECIS binding protein 2-like | 15:49280672-49338760 | 1.85 | 4.65 | 2.51 | 3.20E-02 |
| TOB1 | transducer of ERBB2, 1 | 17:48939583-48987593 | 4.70 | 11.82 | 2.51 | 4.95E-02 |
| OSBPL8 | oxysterol binding protein-like 8 | 12:76745576-76953589 | 2.18 | 5.50 | 2.52 | 2.24E-02 |
| DOCK11 | dedicator of cytokinesis 11 | X:117629860-117820126 | 1.12 | 2.84 | 2.54 | 3.91E-02 |
| SLFN5 | schlafen family member 5 | 17:33570054-33600674 | 2.82 | 7.20 | 2.55 | 1.16E-02 |
| SOCS4 | suppressor of cytokine signaling 4 | 14:55493947-55516206 | 1.00 | 2.54 | 2.55 | 4.50E-02 |
| FGF2 | fibroblast growth factor 2 (basic) | 4:123747862-123844123 | 6.57 | 16.75 | 2.55 | 3.47E-02 |
| SOCS6 | suppressor of cytokine signaling 6 | 18:67956136-67997436 | 0.99 | 2.52 | 2.56 | 2.56E-02 |
| OSGIN2 | oxidative stress induced growth inhibitor family member 2 | 8:90914086-90940116 | 2.57 | 6.60 | 2.57 | 2.04E-02 |
| USP13 | ubiquitin specific peptidase 13 (isopeptidase T-3) | 3:179370542-179507189 | 1.10 | 2.82 | 2.57 | 3.03E-02 |
| C5orf51 | chromosome 5 open reading frame 51 | 5:41904289-41921738 | 1.69 | 4.39 | 2.60 | 2.40E-02 |
| ARHGAP29 | Rho GTPase activating protein 29 | 1:94614543-94740624 | 2.27 | 5.94 | 2.61 | 9.23E-03 |
| DOCK5 | dedicator of cytokinesis 5 | 8:25042237-25275598 | 3.97 | 10.45 | 2.63 | 2.72E-02 |
| GCLM | glutamate-cysteine ligase, modifier subunit | 1:94350760-94374966 | 4.26 | 11.22 | 2.63 | 1.61E-02 |
| MITF | microphthalmia-associated transcription factor | 3:69788585-70017488 | 0.45 | 1.18 | 2.64 | 2.88E-02 |
| NAB1 | NGFI-A binding protein 1 (EGR1 binding protein 1) | 2:191511471-191573442 | 2.03 | 5.35 | 2.64 | 1.16E-02 |
| SIPA1L3 | signal-induced proliferation-associated 1 like 3 | 19:38397867-38699012 | 3.04 | 8.03 | 2.64 | 3.03E-02 |
| FAM86B1 | family with sequence similarity 86, member B1 | 8:12029710-12053789 | 1.12 | 2.97 | 2.66 | 3.37E-02 |
| RRM2B | ribonucleotide reductase M2 B (TP53 inducible) | 8:103216729-103251346 | 4.40 | 11.77 | 2.67 | 9.23E-03 |
| B4GALT5 | UDP-Gal:betaGlcNAc beta 1,4- galactosyltransferase, polypeptide 5 | 20:48249481-48330415 | 8.75 | 23.68 | 2.71 | 2.24E-02 |
| SSX2IP | synovial sarcoma, X breakpoint 2 interacting protein | 1:85109389-85156486 | 1.16 | 3.14 | 2.71 | 9.23E-03 |
| TIMP3 | TIMP metallopeptidase inhibitor 3 | 22:32908538-33454358 | 88.83 | 240.94 | 2.71 | 4.95E-02 |
| AMPD3 | adenosine monophosphate deaminase 3 | 11:10329859-10529126 | 3.79 | 10.29 | 2.71 | 4.35E-02 |
| PBX1 | pre-B-cell leukemia homeobox 1 | 1:164524820-164868533 | 5.63 | 15.35 | 2.72 | 2.24E-02 |
| RPS6KA3 | ribosomal protein S6 kinase, 90kDa, polypeptide 3 | X:20168028-20285523 | 1.39 | 3.81 | 2.74 | 1.16E-02 |
| GARNL3 | GTPase activating Rap/RanGAP domain-like 3 | 9:129986543-130155939 | 0.24 | 0.65 | 2.74 | 4.03E-02 |
| RPL13P12 | ribosomal protein L13 pseudogene 12 | 17:17286690-17287326 | 103.44 | 284.47 | 2.75 | 4.16E-02 |
| PIKFYVE | phosphoinositide kinase, FYVE finger containing | 2:209130990-209223475 | 0.49 | 1.35 | 2.77 | 2.40E-02 |
| ADD3 | adducin 3 (gamma) | 10:111756125-111895323 | 5.21 | 14.60 | 2.80 | 6.70E-03 |
| TNPO1 | transportin 1 | 5:72090231-72212560 | 7.83 | 21.97 | 2.81 | 3.64E-02 |
| PTER | phosphotriesterase related | 10:16478963-16555736 | 0.94 | 2.64 | 2.82 | 4.27E-02 |
| GPR1 | G protein-coupled receptor 1 | 2:207040039-207082771 | 2.67 | 7.54 | 2.82 | 2.40E-02 |
| FIBIN | fin bud initiation factor homolog (zebrafish) | 11:27015627-27018630 | 14.87 | 41.96 | 2.82 | 4.27E-02 |
| FOXF1 | forkhead box F1 | 16:86544132-86548076 | 11.91 | 33.61 | 2.82 | 4.50E-02 |
| GLRB | glycine receptor, beta | 4:157997208-158093242 | 1.00 | 2.82 | 2.83 | 4.72E-02 |
| RALGPS2 | Ral GEF with PH domain and SH3 binding motif 2 | 1:178694299-178889238 | 2.57 | 7.28 | 2.83 | 2.40E-02 |
| ULK2 | unc-51-like kinase 2 (C. elegans) | 17:19674141-19771249 | 2.00 | 5.67 | 2.83 | 2.72E-02 |
| TMEM158 | transmembrane protein 158 (gene/pseudogene) | 3:45265957-45267770 | 42.17 | 119.47 | 2.83 | 3.78E-02 |
| TAF2 | TAF2 RNA polymerase II, TATA box binding protein (TBP)-associated factor, 150kDa | 8:120743014-120845103 | 0.58 | 1.64 | 2.83 | 2.24E-02 |
| SLC4A4 | solute carrier family 4, sodium bicarbonate cotransporter, member 4 | 4:72053002-72437804 | 1.47 | 4.20 | 2.85 | 2.72E-02 |
| AMIGO2 | adhesion molecule with Ig-like domain 2 | 12:47469489-47630443 | 5.26 | 15.14 | 2.88 | 6.70E-03 |
| GFPT2 | glutamine-fructose-6-phosphate transaminase 2 | 5:179727689-179780387 | 4.61 | 13.27 | 2.88 | 4.86E-02 |
| TCF12 | transcription factor 12 | 15:57210820-57591479 | 2.90 | 8.36 | 2.89 | 3.88E-03 |
| KREMEN1 | kringle containing transmembrane protein 1 | 22:29469065-29564321 | 1.45 | 4.19 | 2.89 | 2.40E-02 |
| GAS1 | growth arrest-specific 1 | 9:89559278-89562104 | 30.57 | 88.82 | 2.91 | 2.04E-02 |
| SLC11A2 | solute carrier family 11 (proton-coupled divalent metal ion transporters), member 2 | 12:51373183-51422349 | 4.50 | 13.08 | 2.91 | 9.23E-03 |
| TNRC6B | trinucleotide repeat containing 6B | 22:40440820-40731811 | 0.41 | 1.19 | 2.92 | 2.40E-02 |
| NRIP3 | nuclear receptor interacting protein 3 | 11:9002122-9025596 | 1.77 | 5.16 | 2.92 | 2.04E-02 |
| HRH1 | histamine receptor H1 | 3:11178778-11305243 | 2.51 | 7.35 | 2.93 | 1.37E-02 |
| SLC39A14 | solute carrier family 39 (zinc transporter), member 14 | 8:22224761-22291642 | 14.63 | 42.96 | 2.94 | 6.70E-03 |
| CKAP2L | cytoskeleton associated protein 2-like | 2:113479062-113522254 | 0.42 | 1.23 | 2.95 | 4.35E-02 |
| RIF1 | RAP1 interacting factor homolog (yeast) | 2:152266396-152591001 | 0.46 | 1.38 | 2.98 | 9.23E-03 |
| TTBK2 | tau tubulin kinase 2 | 15:43030931-43213007 | 0.33 | 0.97 | 2.98 | 4.16E-02 |
| SESN2 | sestrin 2 | 1:28586037-28609002 | 6.23 | 18.69 | 3.00 | 6.70E-03 |
| CHN1 | chimerin (chimaerin) 1 | 2:175664090-175870097 | 7.00 | 21.01 | 3.00 | 1.16E-02 |
| SLC36A4 | solute carrier family 36 (proton/amino acid symporter), member 4 | 11:92877340-92931130 | 0.97 | 2.93 | 3.00 | 3.88E-03 |
| ZNF292 | zinc finger protein 292 | 6:87862550-87973914 | 0.35 | 1.06 | 3.00 | 2.24E-02 |
| PAQR3 | progestin and adipoQ receptor family member III | 4:79697495-79860592 | 0.97 | 2.91 | 3.00 | 3.37E-02 |
| RUNX1T1 | runt-related transcription factor 1; translocated to, 1 (cyclin D-related) | 8:92967202-93115514 | 0.26 | 0.79 | 3.02 | 1.16E-02 |
| PTP4A1 | protein tyrosine phosphatase type IVA, member 1 | 6:64231665-64293492 | 14.32 | 43.34 | 3.03 | 6.70E-03 |
| UBN2 | ubinuclein 2 | 7:138915101-138992981 | 0.14 | 0.43 | 3.06 | 3.64E-02 |
| PDE7B | phosphodiesterase 7B | 6:136172833-136546733 | 4.74 | 14.56 | 3.07 | 3.88E-03 |
| IPMK | inositol polyphosphate multikinase | 10:59951277-60027694 | 0.35 | 1.09 | 3.11 | 4.95E-02 |
| DPP4 | dipeptidyl-peptidase 4 | 2:162848750-162931679 | 8.70 | 27.09 | 3.11 | 6.70E-03 |
| SLC7A11 | solute carrier family 7 (anionic amino acid transporter light chain, xc- system), member 11 | 4:138948575-139163503 | 3.85 | 12.01 | 3.12 | 3.88E-03 |
| PMAIP1 | phorbol-12-myristate-13-acetate-induced protein 1 | 18:57567179-57571538 | 1.53 | 4.76 | 3.12 | 2.72E-02 |
| ACAP2 | ArfGAP with coiled-coil, ankyrin repeat and PH domains 2 | 3:194995464-195163807 | 1.04 | 3.27 | 3.13 | 3.88E-03 |
| CD55 | CD55 molecule, decay accelerating factor for complement (Cromer blood group) | 1:207494852-207534311 | 7.53 | 23.62 | 3.14 | 3.03E-02 |
| MAP3K2 | mitogen-activated protein kinase kinase kinase 2 | 2:128056305-128146041 | 0.90 | 2.84 | 3.16 | 4.50E-02 |
| ABCA5 | ATP-binding cassette, sub-family A (ABC1), member 5 | 17:67143354-67323385 | 1.37 | 4.36 | 3.17 | 9.23E-03 |
| PELI1 | pellino E3 ubiquitin protein ligase 1 | 2:64319785-64479993 | 0.51 | 1.64 | 3.19 | 1.61E-02 |
| ANGPT1 | angiopoietin 1 | 8:108261720-108510283 | 0.71 | 2.29 | 3.21 | 1.37E-02 |
| TCF7L2 | transcription factor 7-like 2 (T-cell specific, HMG-box) | 10:114710008-114927437 | 2.35 | 7.60 | 3.23 | 3.88E-03 |
| EGR1 | early growth response 1 | 5:137801178-137805004 | 9.14 | 29.63 | 3.24 | 3.88E-03 |
| KLHL5 | kelch-like 5 (Drosophila) | 4:39046658-39128477 | 6.66 | 21.64 | 3.25 | 3.88E-03 |
| ITGA4 | integrin, alpha 4 (antigen CD49D, alpha 4 subunit of VLA-4 receptor) | 2:182321928-182400914 | 1.11 | 3.64 | 3.26 | 6.70E-03 |
| RAPGEF2 | Rap guanine nucleotide exchange factor (GEF) 2 | 4:160025329-160281321 | 1.10 | 3.62 | 3.28 | 3.88E-03 |
| TRANK1 | tetratricopeptide repeat and ankyrin repeat containing 1 | 3:36868310-36986548 | 0.42 | 1.38 | 3.29 | 4.16E-02 |
| AKAP11 | A kinase (PRKA) anchor protein 11 | 13:42846288-42897396 | 0.66 | 2.20 | 3.32 | 3.88E-03 |
| FCHO2 | FCH domain only 2 | 5:72251807-72386349 | 0.64 | 2.15 | 3.34 | 2.56E-02 |
| MTM1 | myotubularin 1 | X:149737068-149841795 | 0.33 | 1.13 | 3.37 | 4.62E-02 |
| NBPF3 | neuroblastoma breakpoint family, member 3 | 1:21766620-21811498 | 1.43 | 4.86 | 3.40 | 3.88E-03 |
| ZMYM1 | zinc finger, MYM-type 1 | 1:35525386-35581460 | 0.45 | 1.51 | 3.40 | 1.83E-02 |
| QSER1 | glutamine and serine rich 1 | 11:32914723-33014862 | 0.68 | 2.34 | 3.42 | 3.88E-03 |
| NR4A3 | nuclear receptor subfamily 4, group A, member 3 | 9:102584136-102629173 | 1.16 | 4.00 | 3.45 | 1.16E-02 |
| CLMP | CXADR-like membrane protein | 11:122943034-123098985 | 4.75 | 16.38 | 3.45 | 9.23E-03 |
| ARHGAP32 | Rho GTPase activating protein 32 | 11:128834988-129149219 | 0.23 | 0.79 | 3.46 | 2.72E-02 |
| CYP27A1 | cytochrome P450, family 27, subfamily A, polypeptide 1 | 2:219646471-219680016 | 7.94 | 27.45 | 3.46 | 9.23E-03 |
| TANC2 | tetratricopeptide repeat, ankyrin repeat and coiled-coil containing 2 | 17:61086916-61509572 | 0.95 | 3.32 | 3.48 | 1.37E-02 |
| GPR125 | G protein-coupled receptor 125 | 4:22346693-22517677 | 1.77 | 6.16 | 3.49 | 3.88E-03 |
| GGH | gamma-glutamyl hydrolase (conjugase, folylpolygammaglutamyl hydrolase) | 8:63927637-63951730 | 27.75 | 96.91 | 3.49 | 3.88E-03 |
| SMG1 | smg-1 homolog, phosphatidylinositol 3-kinase-related kinase (C. elegans) | 16:18814404-18937776 | 0.82 | 2.87 | 3.49 | 3.88E-03 |
| LURAP1L | leucine rich adaptor protein 1-like | 9:12685438-12822130 | 5.39 | 18.85 | 3.50 | 6.70E-03 |
| IKZF2 | IKAROS family zinc finger 2 (Helios) | 2:213446080-214017151 | 0.14 | 0.50 | 3.51 | 1.83E-02 |
| TMTC4 | transmembrane and tetratricopeptide repeat containing 4 | 13:101256180-101327347 | 1.15 | 4.04 | 3.51 | 3.88E-03 |
| MDFIC | MyoD family inhibitor domain containing | 7:114562208-114659256 | 3.33 | 11.85 | 3.55 | 3.88E-03 |
| ZC3H12C | zinc finger CCCH-type containing 12C | 11:109964086-110042566 | 0.32 | 1.15 | 3.60 | 9.23E-03 |
| GALNTL2 | UDP-N-acetyl-alpha-D-galactosamine:polypeptide N-acetylgalactosaminyltransferase-like 2 | 3:16216155-16273499 | 3.59 | 12.95 | 3.61 | 2.72E-02 |
| CDH2 | cadherin 2, type 1, N-cadherin (neuronal) | 18:25530929-25757410 | 2.71 | 9.81 | 3.62 | 3.88E-03 |
| GPR126 | G protein-coupled receptor 126 | 6:142622990-142767403 | 0.26 | 0.94 | 3.64 | 3.03E-02 |
| ZNF217 | zinc finger protein 217 | 20:52169308-52251129 | 1.30 | 4.76 | 3.67 | 3.88E-03 |
| CPEB4 | cytoplasmic polyadenylation element binding protein 4 | 5:173315282-173388979 | 0.43 | 1.57 | 3.68 | 6.70E-03 |
| SLC7A8 | solute carrier family 7 (amino acid transporter light chain, L system), member 8 | 14:23594503-23652883 | 1.10 | 4.05 | 3.69 | 2.56E-02 |
| HMMR | hyaluronan-mediated motility receptor (RHAMM) | 5:162887174-162921064 | 0.39 | 1.44 | 3.70 | 4.27E-02 |
| SMPDL3A | sphingomyelin phosphodiesterase, acid-like 3A | 6:123110314-123130865 | 1.28 | 4.74 | 3.70 | 9.23E-03 |
| MAN1A1 | mannosidase, alpha, class 1A, member 1 | 6:119498373-119670926 | 1.21 | 4.50 | 3.71 | 6.70E-03 |
| GDNF | glial cell derived neurotrophic factor | 5:37812778-37839788 | 3.37 | 12.53 | 3.71 | 3.88E-03 |
| SLC7A2 | solute carrier family 7 (cationic amino acid transporter, y+ system), member 2 | 8:17354596-17428082 | 2.10 | 7.80 | 3.72 | 3.88E-03 |
| KITLG | KIT ligand | 12:88885884-88974628 | 2.71 | 10.09 | 3.72 | 3.88E-03 |
| CHAC1 | ChaC, cation transport regulator homolog 1 (E. coli) | 15:41245159-41248710 | 12.79 | 47.65 | 3.73 | 6.70E-03 |
| MAPK10 | mitogen-activated protein kinase 10 | 4:86936275-87515284 | 0.43 | 1.61 | 3.76 | 9.23E-03 |
| KIAA1549 | KIAA1549 | 7:138391039-138666064 | 0.21 | 0.78 | 3.78 | 3.91E-02 |
| ATP2B1 | ATPase, Ca++ transporting, plasma membrane 1 | 12:89954905-90103077 | 2.05 | 7.78 | 3.79 | 3.88E-03 |
| IL11 | interleukin 11 | 19:55875756-55881831 | 19.23 | 73.07 | 3.80 | 3.88E-03 |
| ZNF518A | zinc finger protein 518A | 10:97889471-98031333 | 0.15 | 0.56 | 3.80 | 4.72E-02 |
| FNDC3A | fibronectin type III domain containing 3A | 13:49550047-49783888 | 2.01 | 7.68 | 3.81 | 3.88E-03 |
| SLC4A7 | solute carrier family 4, sodium bicarbonate cotransporter, member 7 | 3:27414213-27525911 | 1.28 | 4.91 | 3.82 | 3.88E-03 |
| C1QTNF1 | C1q and tumor necrosis factor related protein 1 | 17:77015290-77045870 | 9.86 | 37.89 | 3.84 | 3.88E-03 |
| LPP | LIM domain containing preferred translocation partner in lipoma | 3:187871071-188608460 | 2.41 | 9.27 | 3.85 | 3.78E-02 |
| HIF1A | hypoxia inducible factor 1, alpha subunit (basic helix-loop-helix transcription factor) | 14:62147758-62263146 | 12.11 | 46.71 | 3.86 | 6.70E-03 |
| ABI3BP | ABI family, member 3 (NESH) binding protein | 3:100467999-100712359 | 17.20 | 66.56 | 3.87 | 3.88E-03 |
| TRIB3 | tribbles homolog 3 (Drosophila) | 20:361260-378203 | 36.85 | 143.47 | 3.89 | 6.70E-03 |
| SEMA3C | sema domain, immunoglobulin domain (Ig), short basic domain, secreted, (semaphorin) 3C | 7:80371853-80551675 | 1.95 | 7.58 | 3.90 | 3.88E-03 |
| TMEM65 | transmembrane protein 65 | 8:125324230-125384933 | 0.79 | 3.11 | 3.94 | 1.16E-02 |
| ABCA1 | ATP-binding cassette, sub-family A (ABC1), member 1 | 9:107543282-107691173 | 0.92 | 3.63 | 3.94 | 3.88E-03 |
| NEDD4 | neural precursor cell expressed, developmentally down-regulated 4, E3 ubiquitin protein ligase | 15:56119119-56285944 | 1.97 | 7.84 | 3.97 | 3.88E-03 |
| DOCK4 | dedicator of cytokinesis 4 | 7:111366173-111846466 | 0.15 | 0.62 | 3.99 | 1.37E-02 |
| CYTL1 | cytokine-like 1 | 4:5016312-5021199 | 4.98 | 19.90 | 4.00 | 3.88E-03 |
| HACE1 | HECT domain and ankyrin repeat containing E3 ubiquitin protein ligase 1 | 6:105175967-105307794 | 0.82 | 3.31 | 4.03 | 3.88E-03 |
| CDK17 | cyclin-dependent kinase 17 | 12:96672038-96794338 | 2.87 | 11.58 | 4.04 | 3.88E-03 |
| IRS2 | insulin receptor substrate 2 | 13:110406183-110438915 | 1.93 | 7.93 | 4.11 | 3.88E-03 |
| BTAF1 | BTAF1 RNA polymerase II, B-TFIID transcription factor-associated, 170kDa (Mot1 homolog, S. cerevisiae) | 10:93683525-93790082 | 0.49 | 2.00 | 4.12 | 3.88E-03 |
| TNFRSF11B | tumor necrosis factor receptor superfamily, member 11b | 8:119935795-119964439 | 48.15 | 204.85 | 4.25 | 3.88E-03 |
| SCG2 | secretogranin II | 2:224461657-224467221 | 2.95 | 12.68 | 4.30 | 3.88E-03 |
| NBEAL1 | neurobeachin-like 1 | 2:203879601-204091101 | 1.35 | 5.84 | 4.32 | 2.88E-02 |
| EFNA5 | ephrin-A5 | 5:106712589-107006596 | 0.65 | 2.81 | 4.32 | 3.88E-03 |
| PLA2G4A | phospholipase A2, group IVA (cytosolic, calcium-dependent) | 1:186798084-186958113 | 3.42 | 14.91 | 4.36 | 3.88E-03 |
| TNFAIP3 | tumor necrosis factor, alpha-induced protein 3 | 6:138178422-138204449 | 2.60 | 11.34 | 4.37 | 3.88E-03 |
| RMRP | RNA component of mitochondrial RNA processing endoribonuclease | 9:35657750-35658014 | 106.73 | 470.62 | 4.41 | 2.56E-02 |
| ACACB | acetyl-CoA carboxylase beta | 12:109549022-109706031 | 0.11 | 0.48 | 4.42 | 2.88E-02 |
| ELTD1 | EGF, latrophilin and seven transmembrane domain containing 1 | 1:79355448-79472403 | 3.61 | 16.13 | 4.47 | 6.70E-03 |
| SGK1 | serum/glucocorticoid regulated kinase 1 | 6:134490383-134639250 | 14.13 | 63.62 | 4.50 | 3.88E-03 |
| IL1R1 | interleukin 1 receptor, type I | 2:102681003-102798568 | 6.06 | 27.83 | 4.60 | 3.88E-03 |
| WNT5A | wingless-type MMTV integration site family, member 5A | 3:55499742-55523973 | 13.51 | 62.55 | 4.63 | 3.88E-03 |
| HIPK2 | homeodomain interacting protein kinase 2 | 7:139246315-139720125 | 1.31 | 6.07 | 4.64 | 3.88E-03 |
| ZEB1 | zinc finger E-box binding homeobox 1 | 10:31596657-31818742 | 1.96 | 9.12 | 4.64 | 3.88E-03 |
| ITPR2 | inositol 1,4,5-trisphosphate receptor, type 2 | 12:26272958-26986131 | 0.51 | 2.39 | 4.68 | 4.62E-02 |
| C5 | complement component 5 | 9:123714615-123812554 | 0.33 | 1.54 | 4.69 | 9.23E-03 |
| CCDC71L | coiled-coil domain containing 71-like | 7:106012696-106410653 | 8.88 | 41.98 | 4.73 | 3.88E-03 |
| PAPPA | pregnancy-associated plasma protein A, pappalysin 1 | 9:118916082-119164601 | 1.80 | 8.55 | 4.76 | 3.88E-03 |
| TWIST2 | twist homolog 2 (Drosophila) | 2:239756672-239795893 | 12.77 | 63.57 | 4.98 | 3.88E-03 |
| GLIS3 | GLIS family zinc finger 3 | 9:3824126-4348392 | 1.09 | 5.43 | 5.00 | 3.88E-03 |
| ZNF765 | zinc finger protein 765 | 19:53868945-53930574 | 0.24 | 1.21 | 5.01 | 3.47E-02 |
| RPLP0P2 | ribosomal protein, large, P0 pseudogene 2 | 11:61382507-61406921 | 0.95 | 5.11 | 5.38 | 3.91E-02 |
| TNC | tenascin C | 9:117782805-117880536 | 6.64 | 35.78 | 5.39 | 3.88E-03 |
| LCOR | ligand dependent nuclear receptor corepressor | 10:98592016-98740800 | 0.21 | 1.14 | 5.49 | 3.88E-03 |
| SLC22A15 | solute carrier family 22, member 15 | 1:116519118-116612675 | 0.27 | 1.47 | 5.49 | 3.88E-03 |
| PDE4D | phosphodiesterase 4D, cAMP-specific | 5:58264864-59843484 | 0.43 | 2.39 | 5.58 | 1.61E-02 |
| MAP3K14-AS1 | MAP3K14 antisense RNA 1 | 17:43268297-43394414 | 0.10 | 0.59 | 5.64 | 3.03E-02 |
| MOCOS | molybdenum cofactor sulfurase | 18:33767481-33852120 | 2.64 | 14.92 | 5.65 | 3.88E-03 |
| CLDN1 | claudin 1 | 3:190023489-190040264 | 0.39 | 2.21 | 5.68 | 3.88E-03 |
| EEF1A1P5 | eukaryotic translation elongation factor 1 alpha 1 pseudogene 5 | 9:135894815-135896553 | 10.56 | 60.44 | 5.72 | 3.88E-03 |
| PLSCR4 | phospholipid scramblase 4 | 3:145910125-145968966 | 1.69 | 9.67 | 5.73 | 3.88E-03 |
| DDR2 | discoidin domain receptor tyrosine kinase 2 | 1:162601162-162750237 | 1.99 | 11.50 | 5.77 | 3.88E-03 |
| ADAMTS6 | ADAM metallopeptidase with thrombospondin type 1 motif, 6 | 5:64444562-64777747 | 0.69 | 4.07 | 5.88 | 3.88E-03 |
| FKBP9L | FK506 binding protein 9-like | 7:55748766-55780945 | 0.23 | 1.36 | 5.88 | 2.56E-02 |
| NR4A2 | nuclear receptor subfamily 4, group A, member 2 | 2:157180943-157198860 | 0.24 | 1.47 | 6.05 | 3.47E-02 |
| IRAK3 | interleukin-1 receptor-associated kinase 3 | 12:66582658-66651214 | 0.16 | 1.03 | 6.27 | 3.88E-03 |
| SPATA13 | spermatogenesis associated 13 | 13:24734860-24896673 | 0.15 | 0.98 | 6.35 | 3.88E-03 |
| PGAP1 | post-GPI attachment to proteins 1 | 2:197699907-197792520 | 0.13 | 0.86 | 6.50 | 3.88E-03 |
| SCN8A | sodium channel, voltage gated, type VIII, alpha subunit | 12:51984049-52202297 | 0.21 | 1.40 | 6.60 | 1.37E-02 |
| PION | pigeon homolog (Drosophila) | 7:76139744-77046115 | 0.38 | 2.73 | 7.14 | 2.24E-02 |
| SLC16A7 | solute carrier family 16, member 7 (monocarboxylic acid transporter 2) | 12:59989847-60176395 | 0.20 | 1.41 | 7.18 | 3.88E-03 |
| STC1 | stanniocalcin 1 | 8:23699427-23712320 | 3.78 | 27.66 | 7.32 | 3.88E-03 |
| MTX3 | metaxin 3 | 5:79275583-79287082 | 0.12 | 0.93 | 7.50 | 6.70E-03 |
| GDF15 | growth differentiation factor 15 | 19:18485540-18499987 | 58.10 | 440.55 | 7.58 | 3.88E-03 |
| SATB1 | SATB homeobox 1 | 3:17199898-18959184 | 0.09 | 0.71 | 7.76 | 9.23E-03 |
| ZFHX4 | zinc finger homeobox 4 | 8:77318888-77779521 | 0.48 | 3.79 | 7.83 | 3.88E-03 |
| PLEKHH2 | pleckstrin homology domain containing, family H (with MyTH4 domain) member 2 | 2:43864411-43995126 | 0.07 | 0.52 | 7.90 | 1.37E-02 |
| NDNF | neuron-derived neurotrophic factor | 4:121956767-122001631 | 0.46 | 3.69 | 8.02 | 2.56E-02 |
| DDX6 | DEAD (Asp-Glu-Ala-Asp) box helicase 6 | 11:118575315-118661858 | 0.70 | 5.61 | 8.06 | 3.88E-03 |
| PTPN14 | protein tyrosine phosphatase, non-receptor type 14 | 1:214517514-214725792 | 0.75 | 6.27 | 8.37 | 3.88E-03 |
| LRRK2 | leucine-rich repeat kinase 2 | 12:40579810-40763087 | 0.07 | 0.58 | 8.60 | 6.70E-03 |
| PTGFR | prostaglandin F receptor (FP) | 1:78695282-79005434 | 0.40 | 3.50 | 8.72 | 3.88E-03 |
| SLC47A1 | solute carrier family 47, member 1 | 17:19398697-19501235 | 0.07 | 0.57 | 8.73 | 3.88E-03 |
| IL1B | interleukin 1, beta | 2:113587327-113594480 | 8.99 | 80.74 | 8.98 | 3.88E-03 |
| FTH1P8 | ferritin, heavy polypeptide 1 pseudogene 8 | X:147133752-147134266 | 1.56 | 14.42 | 9.27 | 2.88E-02 |
| FTH1P11 | ferritin, heavy polypeptide 1 pseudogene 11 | 8:82351670-82445510 | 8.09 | 77.13 | 9.54 | 3.88E-03 |
| RNA5-8SP6 | RNA, 5.8S ribosomal pseudogene 6 | Y:10037763-10037915 | 2098.79 | 20220.80 | 9.63 | 1.16E-02 |
| MME | membrane metallo-endopeptidase | 3:154741912-154901497 | 3.76 | 36.45 | 9.68 | 3.88E-03 |
| NAMPT | nicotinamide phosphoribosyltransferase | 7:105888730-105926772 | 3.67 | 36.39 | 9.91 | 3.88E-03 |
| SMAD1 | SMAD family member 1 | 4:146402345-146479231 | 0.16 | 1.67 | 10.34 | 3.88E-03 |
| ALX4 | ALX homeobox 4 | 11:44281993-44331716 | 0.11 | 1.24 | 11.26 | 2.88E-02 |
| SYT7 | synaptotagmin VII | 11:61282784-61348620 | 0.15 | 1.94 | 12.93 | 3.88E-03 |
| UNC5B | unc-5 homolog B (C. elegans) | 10:72972326-73062621 | 0.44 | 6.12 | 13.77 | 3.88E-03 |
| KCNJ2 | potassium inwardly-rectifying channel, subfamily J, member 2 | 17:68163101-68176189 | 0.26 | 3.78 | 14.72 | 3.88E-03 |
| FSIP2 | fibrous sheath interacting protein 2 | 2:186584600-186698017 | 0.06 | 0.82 | 14.85 | 3.88E-03 |
| IL1A | interleukin 1, alpha | 2:113531491-113542167 | 0.69 | 11.02 | 16.04 | 1.37E-02 |
| CXCL5 | chemokine (C-X-C motif) ligand 5 | 4:74861358-74864496 | 1.45 | 23.55 | 16.22 | 3.88E-03 |
| PTGS2 | prostaglandin-endoperoxide synthase 2 (prostaglandin G/H synthase and cyclooxygenase) | 1:186640922-186649559 | 11.30 | 190.10 | 16.83 | 3.88E-03 |
| NTN1 | netrin 1 | 17:8924858-9147317 | 0.33 | 5.69 | 17.03 | 4.03E-02 |
| CBL | Cbl proto-oncogene, E3 ubiquitin protein ligase | 11:119076751-119177651 | 0.07 | 1.34 | 19.72 | 6.70E-03 |
| LAMP3 | lysosomal-associated membrane protein 3 | 3:182840000-182881627 | 0.07 | 1.44 | 21.08 | 3.88E-03 |
| IL8 | interleukin 8 | 4:74606222-74609433 | 32.00 | 678.70 | 21.21 | 3.88E-03 |
| CXCR7 | chemokine (C-X-C motif) receptor 7 | 2:237476429-237491001 | 0.51 | 10.79 | 21.23 | 3.88E-03 |
| DCST2 | DC-STAMP domain containing 2 | 1:154975126-155006257 | 0.04 | 0.79 | 21.45 | 2.72E-02 |
| SPON1 | spondin 1, extracellular matrix protein | 11:13983913-14295237 | 0.06 | 1.53 | 24.04 | 6.70E-03 |
| TPD52 | tumor protein D52 | 8:80830951-81143467 | 0.01 | 0.34 | 29.35 | 6.70E-03 |
| SLC39A8 | solute carrier family 39 (zinc transporter), member 8 | 4:103172197-103371167 | 0.97 | 29.35 | 30.30 | 3.88E-03 |
| TNFAIP6 | tumor necrosis factor, alpha-induced protein 6 | 2:152214105-152236560 | 0.46 | 38.60 | 83.83 | 4.35E-02 |
| CDR1 | cerebellar degeneration-related protein 1, 34kDa | X:139865424-139866723 | 0.29 | 31.82 | 110.58 | 3.88E-03 |

Supplemental Table 2. List of differentially expressed coding RNAs in Keratoconus-derived human corneal fibroblast vs. normal controls with expression > 20 normalized reads in either cases or controls.

| **Gene name** | **Gene Description** | **Locus** | **Normalized Average expression** | | **Fold change** | **FDR value** |
| --- | --- | --- | --- | --- | --- | --- |
|  |  |  | **Controls (n=5)** | **KC (n=4)** |  |  |
| ANKRD1 | ankyrin repeat domain 1 (cardiac muscle) | 10:92671852-92681033 | 119.62 | 0.28 | -428.03 | 3.88E-03 |
| CRYAB | crystallin, alpha B | 11:111779288-111797596 | 1632.04 | 14.65 | -111.37 | 3.88E-03 |
| OXTR | oxytocin receptor | 3:8661085-9005457 | 52.23 | 0.65 | -80.61 | 9.23E-03 |
| ACTA2 | actin, alpha 2, smooth muscle, aorta | 10:90639490-90775542 | 4845.11 | 65.95 | -73.47 | 3.88E-03 |
| PDLIM3 | PDZ and LIM domain 3 | 4:186422902-186456766 | 20.99 | 0.46 | -45.58 | 3.88E-03 |
| ITGA7 | integrin, alpha 7 | 12:56075329-56118489 | 91.90 | 2.11 | -43.46 | 3.47E-02 |
| MCAM | melanoma cell adhesion molecule | 11:119179240-119191799 | 75.51 | 1.74 | -43.32 | 3.88E-03 |
| ID4 | inhibitor of DNA binding 4, dominant negative helix-loop-helix protein | 6:19837616-19840915 | 34.13 | 0.89 | -38.35 | 3.88E-03 |
| LMCD1 | LIM and cysteine-rich domains 1 | 3:7994491-8653610 | 36.38 | 1.00 | -36.34 | 3.88E-03 |
| SGCA | sarcoglycan, alpha (50kDa dystrophin-associated glycoprotein) | 17:48240883-48258539 | 34.86 | 1.03 | -33.81 | 3.88E-03 |
| HSPB7 | heat shock 27kDa protein family, member 7 (cardiovascular) | 1:16340522-16360545 | 117.56 | 3.74 | -31.46 | 3.88E-03 |
| TINAGL1 | tubulointerstitial nephritis antigen-like 1 | 1:32042115-32053288 | 194.17 | 6.41 | -30.30 | 3.88E-03 |
| HAPLN3 | hyaluronan and proteoglycan link protein 3 | 15:89420518-89438857 | 52.94 | 2.10 | -25.26 | 3.88E-03 |
| KRT7 | keratin 7 | 12:52626303-52715182 | 1280.39 | 50.74 | -25.24 | 3.88E-03 |
| NREP | neuronal regeneration related protein homolog (rat) | 5:110831730-111353006 | 148.69 | 7.45 | -19.96 | 6.70E-03 |
| WFDC1 | WAP four-disulfide core domain 1 | 16:84328251-84363450 | 277.41 | 13.92 | -19.92 | 3.88E-03 |
| KRT18 | keratin 18 | 12:53290970-53346686 | 319.77 | 16.31 | -19.60 | 3.88E-03 |
| MGP | matrix Gla protein | 12:14956505-15059520 | 163.02 | 8.35 | -19.52 | 3.88E-03 |
| LMOD1 | leiomodin 1 (smooth muscle) | 1:201862969-201915715 | 28.81 | 1.72 | -16.77 | 3.88E-03 |
| HES4 | hairy and enhancer of split 4 (Drosophila) | 1:934341-935552 | 126.99 | 8.91 | -14.25 | 3.88E-03 |
| OLFML2B | olfactomedin-like 2B | 1:161952981-161993644 | 39.76 | 2.89 | -13.74 | 3.88E-03 |
| CSPG4 | chondroitin sulfate proteoglycan 4 | 15:75966662-76005189 | 22.62 | 1.74 | -12.97 | 3.88E-03 |
| OLFM2 | olfactomedin 2 | 19:9964393-10047228 | 40.11 | 3.24 | -12.39 | 3.88E-03 |
| LIMS2 | LIM and senescent cell antigen-like domains 2 | 2:128395955-128439360 | 189.90 | 16.06 | -11.83 | 3.88E-03 |
| COL3A1 | collagen, type III, alpha 1 | 2:189839045-189877472 | 297.87 | 25.27 | -11.79 | 3.88E-03 |
| S100A4 | S100 calcium binding protein A4 | 1:153516088-153522612 | 3694.78 | 330.84 | -11.17 | 3.88E-03 |
| NOTCH3 | notch 3 | 19:15270443-15311792 | 47.81 | 4.59 | -10.41 | 3.88E-03 |
| CNN1 | calponin 1, basic, smooth muscle | 19:11649531-11661138 | 213.75 | 21.12 | -10.12 | 3.88E-03 |
| BAMBI | BMP and activin membrane-bound inhibitor homolog (Xenopus laevis) | 10:28966270-28971868 | 21.42 | 2.31 | -9.29 | 3.88E-03 |
| CKB | creatine kinase, brain | 14:103985995-103989448 | 105.09 | 11.75 | -8.94 | 3.88E-03 |
| PDGFA | platelet-derived growth factor alpha polypeptide | 7:536894-559933 | 35.58 | 4.38 | -8.13 | 3.88E-03 |
| FHL1 | four and a half LIM domains 1 | X:135229558-135293518 | 207.80 | 25.69 | -8.09 | 3.88E-03 |
| FGF1 | fibroblast growth factor 1 (acidic) | 5:141689991-142077617 | 24.75 | 3.06 | -8.08 | 3.88E-03 |
| PLAC9 | placenta-specific 9 | 10:81891476-81905115 | 151.76 | 19.52 | -7.78 | 3.88E-03 |
| RRAD | Ras-related associated with diabetes | 16:66955581-66959547 | 50.58 | 6.78 | -7.46 | 3.88E-03 |
| IGFBP7 | insulin-like growth factor binding protein 7 | 4:57829535-58071676 | 1256.86 | 171.41 | -7.33 | 6.70E-03 |
| RASL11A | RAS-like, family 11, member A | 13:27844463-27847827 | 33.82 | 4.72 | -7.17 | 3.88E-03 |
| TPPP3 | tubulin polymerization-promoting protein family member 3 | 16:67423711-67427438 | 20.09 | 3.02 | -6.66 | 3.88E-03 |
| PPFIBP2 | PTPRF interacting protein, binding protein 2 (liprin beta 2) | 11:7534528-7678358 | 33.10 | 5.07 | -6.53 | 1.37E-02 |
| LRRC32 | leucine rich repeat containing 32 | 11:76368099-76381791 | 58.64 | 9.19 | -6.38 | 2.04E-02 |
| TRIM47 | tripartite motif containing 47 | 17:73870241-73875627 | 34.37 | 5.52 | -6.23 | 9.23E-03 |
| COX7A1 | cytochrome c oxidase subunit VIIa polypeptide 1 (muscle) | 19:36641823-36643771 | 257.59 | 42.22 | -6.10 | 3.88E-03 |
| ACTG2 | actin, gamma 2, smooth muscle, enteric | 2:74119440-74146992 | 390.54 | 67.36 | -5.80 | 4.16E-02 |
| C7orf10 | chromosome 7 open reading frame 10 | 7:40174574-40900362 | 72.13 | 13.09 | -5.51 | 3.47E-02 |
| GADD45B | growth arrest and DNA-damage-inducible, beta | 19:2476119-2478257 | 77.20 | 14.64 | -5.27 | 3.88E-03 |
| CSRP2 | cysteine and glycine-rich protein 2 | 12:77252002-77274132 | 116.63 | 22.55 | -5.17 | 3.88E-03 |
| SUSD2 | sushi domain containing 2 | 22:24577226-24585078 | 58.18 | 11.41 | -5.10 | 3.88E-03 |
| MFGE8 | milk fat globule-EGF factor 8 protein | 15:89441915-89456642 | 744.13 | 146.76 | -5.07 | 1.83E-02 |
| TPM1 | tropomyosin 1 (alpha) | 15:63334830-63364114 | 458.04 | 91.41 | -5.01 | 1.16E-02 |
| CXXC5 | CXXC finger protein 5 | 5:139026883-139063467 | 37.46 | 7.80 | -4.80 | 3.88E-03 |
| TRPV2 | transient receptor potential cation channel, subfamily V, member 2 | 17:16317837-16340317 | 80.31 | 17.36 | -4.63 | 3.88E-03 |
| COL5A1 | collagen, type V, alpha 1 | 9:137533619-137736686 | 117.63 | 26.97 | -4.36 | 3.88E-03 |
| ISG15 | ISG15 ubiquitin-like modifier | 1:948802-949920 | 519.58 | 119.30 | -4.36 | 3.88E-03 |
| TP53I11 | tumor protein p53 inducible protein 11 | 11:44740941-44972840 | 61.72 | 14.48 | -4.26 | 3.88E-03 |
| HSPB1 | heat shock 27kDa protein 1 | 7:75931860-75933612 | 3288.73 | 780.07 | -4.22 | 9.23E-03 |
| BGN | biglycan | X:152710177-152775012 | 448.03 | 108.32 | -4.14 | 1.83E-02 |
| F10 | coagulation factor X | 13:113777127-113803843 | 25.83 | 6.27 | -4.12 | 3.88E-03 |
| PRRX2 | paired related homeobox 2 | 9:132427919-132484875 | 370.94 | 90.00 | -4.12 | 3.88E-03 |
| TPD52L1 | tumor protein D52-like 1 | 6:125440194-125585553 | 41.00 | 10.11 | -4.06 | 3.88E-03 |
| PLAU | plasminogen activator, urokinase | 10:75668934-75682535 | 42.81 | 10.84 | -3.95 | 3.88E-03 |
| BCAM | basal cell adhesion molecule (Lutheran blood group) | 19:45312327-45324673 | 85.20 | 21.85 | -3.90 | 3.88E-03 |
| SLC1A4 | solute carrier family 1 (glutamate/neutral amino acid transporter), member 4 | 2:65215610-65250999 | 23.27 | 6.11 | -3.81 | 3.88E-03 |
| EHD1 | EH-domain containing 1 | 11:64619113-64655768 | 112.81 | 29.67 | -3.80 | 3.88E-03 |
| SEMA3B | sema domain, immunoglobulin domain (Ig), short basic domain, secreted, (semaphorin) 3B | 3:50304989-50314977 | 23.07 | 6.11 | -3.78 | 9.23E-03 |
| HTRA1 | HtrA serine peptidase 1 | 10:124221040-124274424 | 704.75 | 189.71 | -3.71 | 4.35E-02 |
| SPON2 | spondin 2, extracellular matrix protein | 4:1160719-1202750 | 49.30 | 13.71 | -3.59 | 3.88E-03 |
| SNTA1 | syntrophin, alpha 1 | 20:31995760-32031698 | 55.75 | 15.55 | -3.59 | 3.88E-03 |
| LGALS3BP | lectin, galactoside-binding, soluble, 3 binding protein | 17:76967319-76976191 | 230.76 | 65.53 | -3.52 | 3.88E-03 |
| FBLN2 | fibulin 2 | 3:13573823-13679922 | 26.39 | 7.53 | -3.50 | 4.35E-02 |
| ERRFI1 | ERBB receptor feedback inhibitor 1 | 1:8064463-8086368 | 42.37 | 12.22 | -3.47 | 3.88E-03 |
| CALHM2 | calcium homeostasis modulator 2 | 10:105206542-105222452 | 20.92 | 6.06 | -3.45 | 3.88E-03 |
| SDC2 | syndecan 2 | 8:97505578-97624000 | 99.60 | 29.09 | -3.42 | 1.16E-02 |
| NLRP1 | NLR family, pyrin domain containing 1 | 17:5402746-5522744 | 97.89 | 29.37 | -3.33 | 3.37E-02 |
| A4GALT | alpha 1,4-galactosyltransferase | 22:43088126-43117304 | 27.50 | 8.28 | -3.32 | 6.70E-03 |
| PTPLA | protein tyrosine phosphatase-like (proline instead of catalytic arginine), member A | 10:17631957-17659376 | 42.32 | 13.13 | -3.22 | 2.88E-02 |
| TPST2 | tyrosylprotein sulfotransferase 2 | 22:26921457-26992681 | 52.16 | 16.20 | -3.22 | 3.88E-03 |
| CD97 | CD97 molecule | 19:14491312-14519537 | 56.87 | 18.39 | -3.09 | 3.88E-03 |
| CRIP2 | cysteine-rich protein 2 | 14:105939298-105946499 | 553.88 | 179.88 | -3.08 | 1.37E-02 |
| STC2 | stanniocalcin 2 | 5:172741715-172756506 | 74.33 | 24.45 | -3.04 | 3.78E-02 |
| PDLIM1 | PDZ and LIM domain 1 | 10:96997328-97050781 | 153.04 | 50.77 | -3.01 | 3.88E-03 |
| RTN2 | reticulon 2 | 19:45988546-46005768 | 30.70 | 10.30 | -2.98 | 3.47E-02 |
| MICAL1 | microtubule associated monoxygenase, calponin and LIM domain containing 1 | 6:109765264-109804440 | 57.99 | 19.88 | -2.92 | 3.37E-02 |
| TSC22D3 | TSC22 domain family, member 3 | X:106956450-107020572 | 23.01 | 7.98 | -2.89 | 6.70E-03 |
| GSTT1 | glutathione S-transferase theta 1 | 22:24376132-24384680 | 102.26 | 36.67 | -2.79 | 6.70E-03 |
| ATOH8 | atonal homolog 8 (Drosophila) | 2:85978466-86015189 | 48.95 | 17.68 | -2.77 | 3.88E-03 |
| ITGA3 | integrin, alpha 3 (antigen CD49C, alpha 3 subunit of VLA-3 receptor) | 17:48133331-48167845 | 49.61 | 18.04 | -2.75 | 4.03E-02 |
| TMEM160 | transmembrane protein 160 | 19:47549164-47551888 | 97.12 | 35.45 | -2.74 | 3.47E-02 |
| SLC25A4 | solute carrier family 25 (mitochondrial carrier; adenine nucleotide translocator), member 4 | 4:186064394-186068434 | 55.76 | 20.62 | -2.70 | 1.83E-02 |
| ID3 | inhibitor of DNA binding 3, dominant negative helix-loop-helix protein | 1:23884408-23886285 | 518.02 | 196.76 | -2.63 | 2.40E-02 |
| NID2 | nidogen 2 (osteonidogen) | 14:52471520-52535712 | 24.34 | 9.24 | -2.63 | 2.56E-02 |
| BMP1 | bone morphogenetic protein 1 | 8:22022248-22069839 | 27.93 | 10.71 | -2.61 | 2.40E-02 |
| CD248 | CD248 molecule, endosialin | 11:66080323-66086708 | 208.19 | 80.67 | -2.58 | 3.88E-03 |
| TCEAL3 | transcription elongation factor A (SII)-like 3 | X:102862378-102885881 | 90.42 | 35.73 | -2.53 | 4.35E-02 |
| PDLIM7 | PDZ and LIM domain 7 (enigma) | 5:176910394-176938275 | 332.60 | 139.62 | -2.38 | 3.64E-02 |
| KLHDC3 | kelch domain containing 3 | 6:42981950-42989036 | 54.84 | 23.08 | -2.38 | 4.35E-02 |
| PDHB | pyruvate dehydrogenase (lipoamide) beta | 3:58413356-58419584 | 55.16 | 25.01 | -2.21 | 4.16E-02 |
| BOK | BCL2-related ovarian killer | 2:242483817-242513546 | 65.43 | 30.06 | -2.18 | 4.03E-02 |
| PRKCDBP | protein kinase C, delta binding protein | 11:6340175-6341877 | 359.54 | 165.43 | -2.17 | 4.72E-02 |
| LPAR1 | lysophosphatidic acid receptor 1 | 9:113635542-113800981 | 31.65 | 74.54 | 2.35 | 2.72E-02 |
| TIMP3 | TIMP metallopeptidase inhibitor 3 | 22:32908538-33454358 | 88.83 | 240.94 | 2.71 | 4.95E-02 |
| RPL13P12 | ribosomal protein L13 pseudogene 12 | 17:17286690-17287326 | 103.44 | 284.47 | 2.75 | 4.16E-02 |
| TMEM158 | transmembrane protein 158 (gene/pseudogene) | 3:45265957-45267770 | 42.17 | 119.47 | 2.83 | 3.78E-02 |
| GAS1 | growth arrest-specific 1 | 9:89559278-89562104 | 30.57 | 88.82 | 2.91 | 2.04E-02 |
| GGH | gamma-glutamyl hydrolase (conjugase, folylpolygammaglutamyl hydrolase) | 8:63927637-63951730 | 27.75 | 96.91 | 3.49 | 3.88E-03 |
| TRIB3 | tribbles homolog 3 (Drosophila) | 20:361260-378203 | 36.85 | 143.47 | 3.89 | 6.70E-03 |
| TNFRSF11B | tumor necrosis factor receptor superfamily, member 11b | 8:119935795-119964439 | 48.15 | 204.85 | 4.25 | 3.88E-03 |
| RMRP | RNA component of mitochondrial RNA processing endoribonuclease | 9:35657750-35658014 | 106.73 | 470.62 | 4.41 | 2.56E-02 |
| GDF15 | growth differentiation factor 15 | 19:18485540-18499987 | 58.10 | 440.55 | 7.58 | 3.88E-03 |
| RNA5-8SP6 | RNA, 5.8S ribosomal pseudogene 6 | Y:10037763-10037915 | 2098.79 | 20220.80 | 9.63 | 1.16E-02 |
| IL8 | interleukin 8 | 4:74606222-74609433 | 32.00 | 678.70 | 21.21 | 3.88E-03 |
